# Environmental Risk Assessment for the Active Pharmaceutical Ingredient Mycophenolic Acid in European Surface Waters

**DOI:** 10.1002/etc.4524

**Published:** 2019-09-19

**Authors:** Jürg Oliver Straub, Rik Oldenkamp, Thomas Pfister, Andreas Häner

**Affiliations:** ^1^ Group Safety, Health, and Environmental Protection, F.Hoffmann‐La Roche, Basle Switzerland; ^2^ Department of Environmental Science Radboud University Nijmegen Nijmegen The Netherlands; ^3^ Environment Department University of York, Heslington York United Kingdom

**Keywords:** Mycophenolic acid, Mycophenolate mofetil, Environmental fate, Ecotoxicology, Environmental risk assessment

## Abstract

An environmental risk assessment is presented for mycophenolic acid (MPA), an immunosuppressive pharmaceutical used for prevention of organ rejection, and its prodrug mycophenolate mofetil (MPM). Mycophenolic acid will not significantly adsorb to activated sludge. In activated sludge, ^14^C‐MPA attained >80% degradation, supporting an older environmental fate test with the same compound. Based on *n*‐octanol/water distribution coefficient (log D_OW_) values of 2.28, 0.48, and ≤–1.54 at pH 5, 7, and 9, respectively, MPA is not expected to bioaccumulate. Sales amounts of MPA+MPM in Europe were used to derive predicted environmental concentrations (PECs) in surface waters; PECs were refined by including expected biodegradation in sewage treatment, average drinking water use, and average dilution of the effluents in the receiving waters per country. In addition, the exposure to pharmaceuticals in the environment (ePiE) model was run for 4 European catchments. The PECs were complemented with 110 measured environmental concentrations (MECs), ranging from below the limit of quantitation (<0.001 µg/L) to 0.656 µg/L. Predicted no‐effect concentrations (PNECs) were derived from chronic tests with cyanobacteria, green algae, daphnids, and fish. The comparison of PECs and MECs with the PNECs resulted in a differentiated environmental risk assessment in which the risk ratio of PEC/PNEC or MEC/PNEC was <1 in most cases (mostly >90%), meaning no significant risk, but a potential risk to aquatic organisms in generally <10% of instances. Because this assessment reveals a partial risk, the following questions must be asked: How much risk is acceptable? and Through which measures can this risk be reduced? These questions are all the more important in view of limited alternatives for MPM and MPA and the serious consequences of not using them. *Environ Toxicol Chem* 2019;38:2259–2278. © 2019 The Authors. *Environmental Toxicology and Chemistry* published by Wiley Periodicals, Inc. on behalf of SETAC.

## INTRODUCTION

Pharmaceuticals in the environment have gained increasing scientific and regulatory attention over the past 30+ yr (Straub and Hutchinson 2014; Kümmerer 2016). Detections, mainly in aquatic environmental media, have multiplied with the recent massive development of analytical technology. The biological potency of active pharmaceutical ingredients (APIs) has led to the assumption that residues may cause adverse effects in environmental organisms. Regulatory requirements for environmental risk assessments for APIs have been introduced and developed further in the United States (Center for Drug Evaluation and Research 1998) and the European Union (Straub and Hutchinson 2014), and regulations are expected in the near future in Japan (Japanese Ministry of Health, Labor, and Welfare 2016) and Canada (J. Chateauvert, personal communication). One particular issue regarding pharmaceuticals in the environment concerns those APIs that have carcinogenic, mutagenic, or reprotoxic properties in laboratory mammals, due to the suspicion of comparable adverse effects in environmental organisms (Committee for Medicinal Products for Human Use 2015). The present study is an environmental risk assessment for an API with such carcinogenic, mutagenic, or reprotoxic properties, mycophenolic acid (MPA), which has gathered attention in various prioritization lists for pharmaceuticals in the environment (Roos et al. 2012; Daouk et al. 2015; Guo et al. 2015; Aubakirova et al. 2017; Santos et al. 2017a).

**Figure 1:**
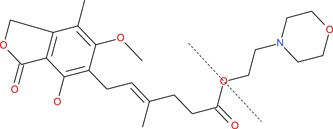
Structure of mycophenolate mofetil (whole molecule), mycophenolic acid (moiety to the left of broken line), and morpholinoethanol (mofetil; moiety to the right of broken line).

Mycophenolic acid is 6‐(1,3‐dihydro‐4‐hydroxy‐6‐methoxy‐7‐methyl‐3‐oxo‐5‐isobenzofuranyl)‐4‐methyl‐4‐hexenoic acid, a small molecule with a molecular weight of 320.34 g/mol (CAS no. 24280–93–1; Roche 2017a; Figure 1). It was discovered >100 yr ago as a natural substance synthesized by various molds of the genus *Penicillium* (Anderson et al. 1988; cited in Noto et al. 1969; Lee et al. 1990). It was recognized early to potentially be a broad‐spectrum API, due to antibacterial, antiviral, antifungal, antipsoriatic, and anticancer activity (the latter through inhibition of angiogenesis; Florey et al. 1946; Cline et al. 1969; Noto et al. 1969; Lee et al. 1990; Silverman et al. 1997; Wu et al. 2006). It was also shown to be a specific inhibitor of the immune system and was developed in the early 1990s as an immunosuppressant API used for the prevention of solid organ rejection in transplant recipients (Fulton and Markham 1996).

Mycophenolic acid is a reversible, potent, and noncompetitive inhibitor of the enzyme inosine‐5′‐monophosphate dehydrogenase (IMPDH), which is essential for the biosynthesis of purines (Wishart et al. 2018; Royal Pharmaceutical Society 2018). Inhibition of IMPDH particularly affects lymphocytes because they rely almost exclusively on de novo purine synthesis, whereas many other cell types can switch to salvaging pathways (Wishart et al. 2018; Royal Pharmaceutical Society 2018). Therefore, MPA suppresses the proliferation of T‐ and B‐lymphocytes and also inhibits antibody formation by B‐lymphocytes (Wishart et al. 2018). This selectivity for lymphocytes explains the off‐label use of MPA in autoimmune diseases. The broad spectrum of activity of MPA (just described) suggests that IMDPH occurs in all eukaryotes (Gunnarsson 2008; Santos et al. 2017b), which is supported by the ECOdrug database (Verbruggen et al. 2017), and also in prokaryotes, in which IMPDH inhibition by MPA seems to be weaker (Digits and Hedstrom 1999; Gunnarsson 2008).

In view of its relatively low oral bioavailability, MPA was derivatized by Syntex (now integrated into F.Hoffmann‐La Roche [Roche]) in the early 1990s to the prodrug mycophenolate mofetil (MPM; CAS no. 128794–94–5, 433.5 g/mol; Figure 1; Roche 2017b), the morpholinoethyl ester of MPA (Lee et al. 1990; Fulton and Markham 1996). Mycophenolate mofetil has 1.3 to 2.36 times higher oral bioavailability than MPA and is rapidly and fully hydrolyzed to MPA in the liver and intestine (Lee et al. 1990; Shipkova et al. 2005). Thus, pharmacologically active MPA levels are reached subsequent to MPM dosage of three‐quarters to less than one‐half of the corresponding amount of MPA.

Due to its mode of action, MPA is also mutagenic and teratogenic in mammals; hence, based on data from the Committee for Medicinal Products for Human Use (2015), concerns exist over persistence, bioaccumulation, or specifically high ecotoxicity properties of MPA in the environment. Some fundamental environmental data were developed by Syntex at the time of clinical development of MPA and MPM, but no chronic ecotoxicity tests or other basic environmental risk assessment data that are considered crucial today (Committee for Medicinal Products for Human Use 2015). Additional tests were commissioned by Roche to allow an updated environmental risk assessment for European surface waters that considers both the potential persistence, bioaccumulation, or high ecotoxicity properties and the total actual use of MPM and MPA; the present study describes the resulting environmental risk assessment.

## MATERIALS AND METHODS

### Existing tests, databases, and literature data

Roche internal databases, the European Chemicals Agency (2018) database, and the scientific literature were searched for physicochemical, toxicological, and environmentally relevant data as well as measured environmental concentrations (MECs) for MPM and MPA. In addition, the IQVIA MIDAS Quantum subscription database (IQVIA 2018) was queried for sales amounts in kilograms of MPM and MPA in European countries for the period 2004 to 2017, with the aim of basing the environmental risk assessment on realistic use data.

The existing Syntex tests comprise the following internal, non–good–laboratory–practice (GLP) tests and external, GLP‐compliant studies: vapor pressure of MPA; photodegradation of MPA in aqueous solutions; and biodegradability of ^14^C‐labeled MPM (2 test substances with radiolabels in different moieties of the molecule) in river water and sediment. Descriptions of the test methods applied are given in the Supplemental Data. Additional tests following Organisation for Economic Co‐operation and Development (OECD; 2018) test guidelines in compliance with GLP, ensuring the validity of the procedures and statistics (Caldwell et al. 2018), were commissioned by Roche. Because MPM is fully cleft to MPA and the excreted metabolites are 7‐O‐MPA glucuronide (MPAG) and MPA, all new tests were performed with the active moiety MPA. The following tests were performed: OECD test guideline 106 phase I batch equilibrium adsorption test (Organisation for Economic Co‐operation and Development 2000); OECD test guideline 314B activated sludge biodegradation simulation test (Organisation for Economic Co‐operation and Development 2008); OECD test guideline 201 algal growth inhibition test with *Anabaena flos‐aquae* (cyanobacteria; Organisation for Economic Co‐operation and Development 2011); OECD test guideline 211 chronic reproduction test with *Daphnia magna* (Organisation for Economic Co‐operation and Development 2012)*;* and a fish partial life cycle test consisting of an OECD test guideline 229 short‐term reproduction test (Organisation for Economic Co‐operation and Development 2012b) followed by an OECD test guideline 210 early life stage test with *Danio rerio* (Organisation for Economic Co‐operation and Development 2013). Also, a ready biodegradability study according to OECD test guideline 301F was performed with MPM (Organisation for Economic Co‐operation and Development 1992). Descriptions of the test methods applied are given in the Supplemental Data.

### Predicted and measured environmental concentrations

For 24 documented European countries, the administered amount of MPA and the stoichiometric fraction of MPA from MPM administration in kg/yr were retrieved for the period 2004 to 2017 (IQVIA 2018), for the derivation of predicted environmental concentrations (PECs). For each combination of country and year, these amounts were summed and divided by the population in that country and year (European Commission 2018), and by 365 d, to derive year‐specific daily per capita uses. In addition to single‐country uses, we also derived year‐specific European uses as the average daily per capita use over all countries.

An initial MPA sewage treatment plant (STP) PEC following the European Medicines Agency (EMA) guideline (Committee for Medicinal Products for Human Use 2015) was obtained by dividing the highest overall European MPA daily per capita use by a default 200 L of wastewater/inhabitant/day; no human metabolism or removal in STPs was included. When the EMA guideline default dilution factor was applied, the initial surface water PEC was 10 times lower (Committee for Medicinal Products for Human Use 2015). (Note that this default has been contested for single German rivers by Link et al. [2017]. On the other hand, data by Keller et al. [2014] suggest that the average and median dilution factors for European countries are >10, whereas Belgium, for example, has a lower dilution.) Refined MPA STP PECs per country were calculated by dividing the highest country‐specific daily per capita MPA use by the country‐specific daily water use (Keller et al. 2014). Refined MPA surface water PECs per country were then determined by deducting a predicted STP removal based on the OECD test guideline 314B (Organisation for Economic Co‐operation and Development 2008) test results using SimpleTreat 4.0 model (Struijs 2015) for 80% of MPA mass (no wastewater treatment was assumed for the remaining 20%) and further division by the median country‐specific dilution factor (Keller et al. 2014). For comparison with the initial PECs based on the EMA guideline, average European refined, population‐, water‐use‐, and dilution‐factor–weighted PECs for STPs and receiving waters were calculated as well.

The geographically based **e**xposure to **p**harmaceuticals **i**n the **e**nvironment (ePiE; Oldenkamp et al. 2018) model was applied to derive distributions of PECs in European river catchments. The ePiE model was developed within the research project **i**ntelligence‐led assessment of **p**harmaceuticals **i**n the **e**nvironment (iPiE 2019) and combines high‐resolution georeferenced information on river flow and locations of STPs with information on number of inhabitants attached, API consumption, human metabolism, and fate of APIs during their passage through STPs and receiving surface waters. The model was run under annual mean, maximum, and minimum monthly flow conditions for the year 2015, and was applied to 4 climatically and hydrologically diverse river basins: the Rhine catchment, with a total population of approximately 58 million in 2009 (Uehlinger et al. 2009), receiving water and effluents from Switzerland, Liechtenstein, Austria, France, Germany, Luxembourg, Belgium, and The Netherlands; the Ouse Basin in northern England, UK; and the Turia and Guadalquivir catchments, in eastern and southern Spain. Average total MPA use data for these countries (except Liechtenstein, for which no separate use data are available) were entered into ePiE, as were physicochemical and environmental fate data, to derive catchment‐wide PECs reflecting the geographical distributions of the modeled concentrations. We regarded the Rhine catchment as representative of climatically moderate northwestern Europe, with a significant share of >10% of the total European population (Uehlinger et al. 2009; European Commission 2018). The 2 Spanish catchments are representative of warmer and drier southern Europe; the Guadalquivir is a basin near the large cities of Cordoba and Seville, with a correspondingly high population/STP effluent contribution; the Turia catchment is smaller and more agriculturally influenced. Finally, the Ouse catchment is regarded as representative for more Atlantic‐influenced western Europe.

The MECs for MPA were searched for in the scientific literature using the terms “mycophenol*”, “environment*”, and “concentration”, collated and percentage‐ranked following Straub (2008).

### Predicted no‐effect concentrations

The predicted no‐effect concentration (PNEC) for STPs (PNEC_STP_) was derived by dividing the lowest activated sludge respiration inhibition no‐observed‐effect concentration (NOEC) by an assessment factor of 10. The surface (fresh) water PNEC_SW_ was derived in 2 ways, from the lowest chronic NOEC on the one hand and from the lowest chronic 10% effect concentration (EC10) on the other, from (sub)chronic tests with fish, daphnids, green algae, and cyanobacteria (for the latter 2 the NOErC/ErC10 for growth rate [r] was used), using an assessment factor of 10 (Committee for Medicinal Products for Human Use 2015).

### Risk assessment

The potential risk for STPs and surface freshwater organisms was characterized by dividing the various PECs and MECs by the respective PNECs for MPA (Committee for Medicinal Products for Human Use 2015). Further risk assessments were performed for antibiotic resistance risk due to the described antibiotic activity of MPA, risk for environmental top predators, and also risk for humans through so‐called secondary poisoning from uptake of water or fish.

Antibiotic resistance risk is assessed through comparing PECs or MECs with a PNEC for formation or maintenance of antibiotic resistance (PNEC_ABR_). There is no universally accepted or regulatory algorithm for deriving a reliable PNEC_ABR_ for bacteria, so for the time being this is approximated by applying assessment factors to bacterial minimal inhibitory concentrations (MICs). Kümmerer and Henninger (2003) applied an assessment factor of 100 to the lowest bacterial MIC/antibiotic. In 2016, Bengtsson‐Palme and Larsson used the lower 1st percentile (%ile) MIC/antibiotic and applied 2 assessment factors, a general factor of 10, and an additional factor that depends on the size of the MIC dataset and reflects the magnitude of uncertainty. To be fully consistent with Bengtsson‐Palme and Larsson's (2016) approach to PNEC_ABR_ derivation, the authors were contacted for help; they (J. Bengtsson‐Palme, Department of Infectious Diseases, Institute of Biomedicine, The Sahlgrenska Academy, University of Gothenberg, Gothenberg, Sweden, personal communication) state that “we only use species with 10 or more MIC observations, to avoid drawing conclusions from insufficient data [...]. The only species with >10 observations in the Noto et al. (1969) paper is *S[taphylococcus] aureus*. In addition, when the MIC1% are selected, *only one MIC* [J.O.Straub's italics] value is selected per species (the lowest). Finally, the uncertainty factor for low sampling numbers is calculated as: (observed lowest MIC) × (number of tested species) ÷ 41.” Both these provisional PNEC_ABR_ values were derived from the MPA MICs; the lower was selected and compared with the PECs and MECs.

The potential risk for (semi)aquatic top predators such as otters that consume fish and drink surface water was assessed by maximum tolerable daily intake (MTDI) calculated following Murray‐Smith et al. (2012) and the European Union technical guidance for deriving environmental quality standards (European Commission 2011). In brief, acute and long‐term mammalian toxicity results are divided by appropriate assessment factors, depending on the nature and duration of the tests, to derive an MTDI. For an otter, a default body mass of 10 kg, an intake of 1 kg fish, and 0.79 L of water/d is assumed; the concentration of the substance in fish results from the surface water PEC and the bioconcentration factor (BCF). The combined daily intake from fish and water is then compared with the MTDI (Murray‐Smith et al. 2012).

Similarly, potential risk for humans from secondary poisoning is assessed by using the acceptable daily exposure (ADE) value for MPA (T. Pfister, unpublished data), which considers toxicological data as well as human experience and covers all routes of uptake, including sensitive subpopulations, over 365 d/annum, with the combined worst‐case uptake through drinking water and fish. No removal of MPA in drinking water production is assumed, but a default intake of 2 L water and 115 g fish/person and day is assumed (European Commission 2011; Murray‐Smith et al. 2012). This combined uptake is then compared with the ADE.

## RESULTS AND THEIR CONTEXT

### Physicochemical properties and environmental fate

Syntex derived many basic physicochemical properties for both MPM and MPA in the early 1990s. The results for MPA are mostly given in the present study. Syntex internal tests were performed without GLP compliance, but with full documentation (protocol, experimental data, analytics with validation, and calculation algorithms included in reports): solubility in buffered aqueous solutions, dissociation constant (p*K*_a_) determined by titration, screening vapor pressure, *n*‐octanol/water distribution coefficient (log *D*_OW_) by the shake‐flask method, and screening hydrolysis (Table 1; T.J. Lynch and N. Licato, Syntex, Palo Alto, CA, USA/F.Hoffmann‐La Roche, Basle, Switzerland, unpublished data; V. Nicholson, Syntex, Palo Alto, CA, USA/F.Hoffmann‐La Roche, Basle, Switzerland, unpublished data; V. Nicholson and N. Licato, Syntex, Palo Alto, CA, USA/F.Hoffmann‐La Roche, Basle, Switzerland, unpublished data; V. Nicholson et al., Syntex, Palo Alto, CA, USA/F.Hoffmann‐La Roche, Basle, Switzerland, unpublished data; A. Young and N. Licato, Syntex, Palo Alto, CA, USA/F.Hoffmann‐La Roche, Basle, Switzerland, unpublished data).

**Table 1:** Physicochemical data for mycophenolic acid^a^

Property	Value	Unit	Condition	Reference
Solubility in water	45	mg/L	pH 5, 25 ± 2 °C	T.J. Lynch and N. Licato, Syntex, Palo Alto, CA, USA/F.Hoffmann‐La Roche, Basle, Switzerland, unpublished data.
	710	mg/L	pH 7, 25 ± 2 °C	T.J. Lynch and N. Licato, unpublished data.
p*K*_a_, carboxylic acid	4.58	—	25 ± 2 °C	T.J. Lynch and N. Licato, unpublished data.
p*K*_a_, phenolic	8.045	—	25 ± 2 °C	V. Nicholson and N. Licato, Syntex, Palo Alto, CA, USA/F.Hoffmann‐La Roche, Basle, Switzerland, unpublished data.
Vapor pressure	3.2 × 10^–7^	Torr		V. Nicholson et al., Syntex, Palo Alto, CA, USA/F.Hoffmann‐La Roche, Basle, Switzerland, unpublished data.
	= 4.27^–7^	hPa		V. Nicholson et al., unpublished data.
Log *D*_OW_	2.28	—	pH 5	A. Young and N. Licato, Syntex, Palo Alto, CA, USA/F.Hoffmann‐La Roche, Basle, Switzerland, unpublished data.
	0.48	—	pH 7	A. Young and N. Licato, unpublished data.
	≤–1.54	—	pH 9	A. Young and N. Licato, unpublished data.
Screening hydrolysis	46.15	% Substance loss	pH 5, 50 °C, 5 d	A. Young and N. Licato, unpublished data.
	29.03	% Substance loss	pH 7, 50 °C, 5 d	A. Young and N. Licato, unpublished data.
	36.67	% Substance loss	pH 9, 50 °C, 5 d	A. Young and N. Licato, unpublished data.

^a^ Based on tests by Syntex. Test procedures are described in the Supplemental Data.

The log *D*_OW_ values ≤2.28 at pH 5 to 9 (A. Young and N. Licato, unpublished data) suggest that MPA will not bioaccumulate significantly. Because no experimental bioaccumulation data have been located, quantitative structure–property (QSPR) models were used to estimate BCFs for MPA. The EPISuite Ver 4.11 software calculates a BCF (wet wt) for fish of 3.16, independent of ionization (US Environmental Protection Agency 2016). The SciFinder database models BCFs along most of the pH range, with the highest value of 486 at pH 1, which drops to 161 at pH 5, to 23.2 at pH 6, to 2.49 at pH 7, and to 1.0 at pH 8 to 10 (American Chemical Society 2018), echoing the log *D*_OW_ distribution (Table 1). The geometric average of the SciFinder values in the environmentally relevant range of pH 5 to 9 is a BCF of 6.22. These models support the log *D*_OW_‐based prediction of no significant bioaccumulation for MPA.

The low vapor pressure of <10^–6^ hPa (V. Nicholson and N. Licato, unpublished data) suggests that MPA will not volatilize to a significant extent. The screening hydrolysis test resulted in 29.03 to 46.15% substance loss at pH 5 to 9 (A. Young and N. Licato, unpublished data). These losses suggest that under environmental conditions (default 12 °C water temperature in Europe) there will only be slow hydrolysis. In confirmation, Franquet‐Griell et al. (2017a) recently reported a hydrolytic degradation constant for MPA of 0.0002 min^–1^.

The photodegradation test shows *k*_photo_ values at pH 5 ranging from 0.0017 min^–1^ in winter to 0.0059 min^–1^ in summer, at pH 7 from 0.0049 min^–1^ to 0.018 min^–1^ and at pH 9 from 0.0087 min^–1^ to 0.031 min^–1^ (V. Nicholson, unpublished data). Marín‐García (2015) studied ultraviolet (UV)‐C photodegradation of MPA; at pH 7, 20 mg/L MPA showed negligible photodegradation but higher rates at pH 10 to 12 (Marín‐Garcia 2015), suggesting that very‐short‐wave UV‐C does not significantly degrade MPA at environmentally relevant pH levels. In contrast, Franquet‐Griell et al. (2017a) reported MPA *k*_photo_ values of 0.0284 min^–1^ for UV‐C irradiation and 0.004 min^–1^ in a Solar Box artificial sunlight reactor. The former rate suggests relatively rapid degradation under UV‐C exposure, whereas the latter rate compares rather well with the data just given above developed under natural sunlight by V. Nicholson (unpublished data). Giebułtowicz and Nałęcz‐Jawecki (2016) presented a UV‐visual light spectrum of MPA in their Supporting Information with a λ_max_ at 215 nm and 2 sequentially lower peaks at 248 and 297 nm, confirming UV‐C photosensitivity. Altogether, these data suggest a notable removal of MPA in surface waters through photodegradation, which, however, is strongly dependent on season, latitude, water depth, turbidity, and pH level.

The sediment/water fate test (Z. Yan, ABC Laboratories, Columbia MO, USA, on behalf of Syntex, Palo Alto, CA, USA/F.Hoffmann‐La Roche, Basle, Switzerland, unpublished data) shows that the overall ^14^C mass balance for all systems and concentrations was satisfactory, with a minimum of 90.4 ± 0.9% and a maximum of 99.9 ± 5.6%. At the end of the test, in the carboxyl‐^14^C‐labeled MPM systems, 58.9 and 60.2% (for 1 and 5 mg/L dosing, respectively) of applied ^14^C activity was trapped as ^14^CO_2_, whereas 4.8 and 6.7% remained in water and 17.6 and 20.3% was bound to sediment, plus some activity found in the samples for analyses. In the morpholine‐^14^C–labeled MPM systems, 21.5 and 28.0% was trapped as ^14^CO_2_, whereas 41.5 and 32.4% remained in the water and 12.0 and 12.4% was bound to sediment, plus the samples activity. The analysis of ^14^CO_2_ and high‐performance liquid chromatography (HPLC) data showed that full primary degradation took place in all systems and dosings within 7 d, resulting in several transformation products. The transformation products were further biodegraded, partially mineralized, or bound to sediment during the 64‐d test period. HPLC cochromatography of day 64 samples with the respective ^14^C reference standards showed that both forms of ^14^C‐labelled MPM had completely disappeared, leaving no significant peak at all in the carboxyl‐^14^C‐MPM systems and a small peak of an unidentified transformation product in the morpholine‐^14^C‐MPM systems. This particular transformation product was shown by HPLC to reach a peak of approximately 75 to 85% relative to initially applied by day 7 and then to decrease to 40 to 50% in the morpholine‐^14^C‐MPM systems, and to 5 to 10% in the carboxyl‐^14^C‐MPM systems. The report states, “Compared to the morpholine‐^14^C radiolabeled metabolite, the carboxyl‐^14^C radiolabeled metabolite (probably ^14^C‐mycophenolic acid) is much more easily biodegraded and mineralized” (Z. Yan, unpublished data, p 23). This sediment/water fate test shows that the parent MPM is lost from the systems with a 50% disappearance time (DT50) of <2 d in all cases. The carboxyl‐^14^C‐MPM transformation product, tentatively identified by the authors as ^14^C‐MPA, is nearly completely degraded by the end of the test, with a DT50 of approximately 14 d. The unidentified morpholine‐^14^C‐MPM transformation product is degraded much more slowly, but on day 64 this transformation product, the only peak remaining, had a much smaller area (no quantitative data available) on the chromatogram, compared with the reference standard. Altogether, this test suggests that MPM is transformed rapidly in natural river water/sediment systems, and may in part form nonextractable, bound sediment residues while the remainder in the aqueous phase is partially to nearly fully mineralized.

Franquet‐Griell et al. (2017a) also investigated the biodegradation of MPA in an aerobic sequencing batch reactor with an initial concentration of 1 to 1.2 g/L activated sludge that was run for 5 cycles of 48 h each, after which the activated sludge was left to settle, the supernatant was decanted, and each cycle was supplied with fresh primary effluent spiked with the same MPA concentration. While in the first cycle, approximately 58% of MPA was still detected after 48 h; in the fifth cycle, no MPA at all was detected already after 24 h, showing the adaptation of the biomass to degrade MPA. Franquet‐Griell et al. (2017a) calculated biodegradation rate constants *k*_biodeg_ for MPA in the first cycle of 0.0017 min^–1^ and in the fifth cycle of 0.006 min^–1^, specifically remarking about the “degradation capacity of aerobic activated sludge for [this] compound.”

The new adsorption test following OECD test guideline 106 (Organisation for Economic Co‐operation and Development 2000) showed satisfactory mass balances between 95 and 107%; based on pretests, MPA was shown by analytics to be stable over the selected equilibration time of 10 h (recoveries from 0.01 M CaCl_2_ after 24 h: 104% at 0.2 mg MPA/L and 97% at 20 mg MPA/L). The main test resulted in soil *K*_d_ values of 2.2, 2.8, and 5.0 L/kg and activated sludge *K*_d_ values of 9.3 and 13 L/kg; normalizing to organic carbon content, the highest *K*_OC_ for MPA was 37 L/kg in one of the activated sludges (V. Halász‐Laky, Toxi‐Coop ZRT, Balatonfüred, Hungary, on behalf of F.Hoffmann‐La Roche, Basle, Switzerland, unpublished data). Hence, MPA does not sorb significantly to activated sludge in STPs, but is mobile and remains in the aqueous phase (Briggs 1973; McCall et al. 1980). Thus, MPA is not expected to be significantly transferred to soil with surplus sludge (Committee for Medicinal Products for Human Use 2015).

An OECD test guideline 301F (Organisation for Economic Co‐operation and Development 1992) ready biodegradability test with MPM at a nominal concentration of 100 mg/L (A. Häner, unpublished data) showed primary degradation by HPLC without oxygen uptake, which was interpreted as hydrolysis of the mofetil ester without subsequent biodegradation of both moieties.

**Figure 2:**
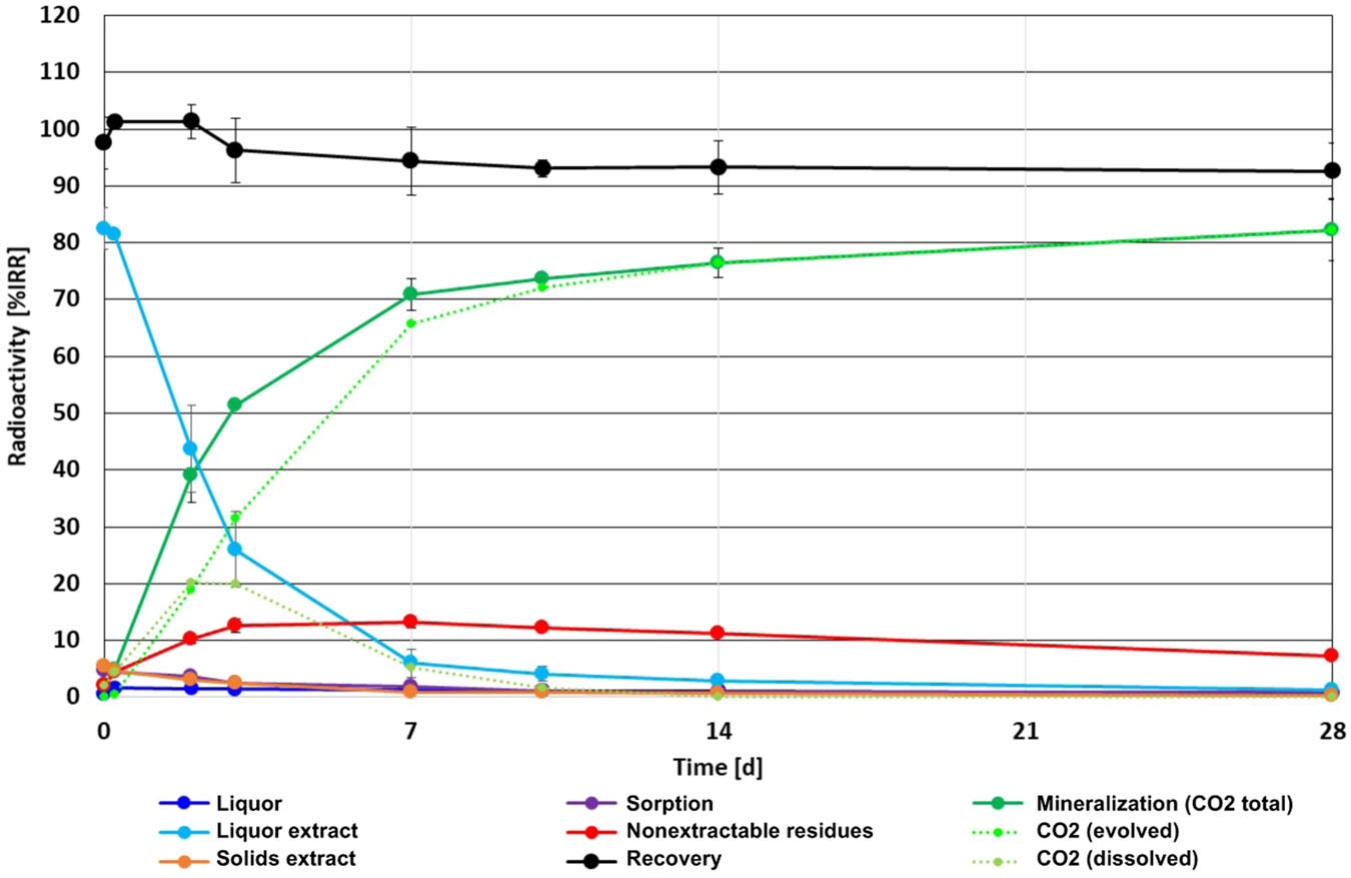
Graph of Organisation for Economic Co‐operation (OECD) test guideline 314B activated sludge biodegradation test with [^14^C]‐mycophenolic acid, ECT Oekotoxikologie on behalf of Roche (Junker T, Herrchen M. 2017. Mycophenolic acid, [carboxyl‐14C]: A study on the biodegradation in activated sludge according to OECD guideline no. 314B: Simulation tests to assess the biodegradability of chemicals discharged in wastewater—Biodegradation in activated sludge. ECT Oekotoxikologie, Flörsheim, Germany, on behalf of F.Hoffmann‐La Roche, Basle, Switzerland, unpublished data). %IRR = percentage of initially recovered radioactivity; NER = nonextractable residues.

The new OECD test guideline 314B (Organisation for Economic Co‐operation and Development 2008) activated sludge degradation test (T. Junker and M. Herrchen, ECT Oekotoxikologie, Flörsheim, Germany, on behalf of F.Hoffmann‐La Roche, Basle, Switzerland, unpublished data) fulfilled all quality and validity criteria (see the Supplemental Data). The test item was rapidly mineralized, with biodegradation starting directly after application, and increasing rapidly at the beginning and more slowly afterward up to 82.2% of initially recovered radioactivity (iRR) at day 28 (Figure 2). Mineralization comprises evolved CO_2_ (detected in the traps) as well as CO_2_ dissolved in the sludge. The latter was highest at day 2 and decreased afterward, whereas the amount of evolved CO_2_ increased continuously until the end of the study. At the start of the test (day 0), 82.5% iRR was detected in the liquor extracts; the liquor extract radioactivity decreased to 1.2% iRR at the end of the test. At no time point was a noteworthy amount of radioactivity (>2% iRR throughout the test period) detected in the liquor after extraction. Radioactivity in the solids extract was highest at day 0 (5.5% iRR) and decreased afterward to 0.3% iRR after 28 d. Nonextractable residues increased at the beginning up to 10.2% iRR on day 2, did not change substantially until day 14 (range: 11.1 to 13.1% iRR), and then fell to 7.3% iRR on day 28.

Using the curve for total CO_2_ produced over time in this OECD test guideline 314B (Organisation for Economic Co‐operation and Development 2008) test (T. Junker and M. Herrchen, unpublished data), a biodegradation rate constant *k*_biodeg_ of 0.0174 h^–1^ or 0.00029 min^–1^ can be calculated for MPA in STPs. Based on this *k*_biodeg_ and physicochemical properties for MPA, SimpleTreat 4.0 (Struijs 2015) calculated a removal of 12% in STPs, mostly through biodegradation (0.27% in the primary settler and 11.5% in the aeration tank), with a small fraction of 0.13% adsorbed to sludge, in agreement with the OECD test guideline106 (Organisation for Economic Co‐operation and Development 2000) adsorption results mentioned in the previous paragraph (V. Halász‐Laky, unpublished data). However, when the *k*_biodeg_ data developed by Franquet‐Griell et al. (2017a) were entered in the sequencing batch reactor for *k*_biodeg_ of 0.0017 min^–1^ in the first cycle, SimpleTreat calculated a removal of 43.6% in STPs (0.272% biodegradation primary settler, 43.24% aeration tank; 0.080% adsorbed); for *k*_biodeg_ in the fifth cycle of 0.006 min^–1^, SimpleTreat calculated a removal of 73.1% in STPs (0.272% primary settler, 72.77% aeration tank; 0.0373% adsorbed; Struijs 2015). A high degradation rate is supported by data from Franquet‐Griell et al. (2017b), who measured STP influents and effluents and compared solid phase extraction and macroporous ceramic passive samplers in Spain. The STP in question treats 65% of the wastewaters from Barcelona and surroundings (2 843 750 inhabitant equivalents) and receives urban waters, effluents from 3 large hospitals, and industrial waters; the STP performs biological treatment without nitrogen and phosphorus removal. Based on substance concentrations in effluent and influent, removal rates of >90% emerged for MPA (Franquet‐Griell et al. 2017b).

As a further confirmation of biodegradability during wastewater treatment, weekly measurements of MPM and MPA concentrations in the influent and effluent of a small industrial activated sludge STP at a Roche production site were made during 8 wk of MPM production in 2008 (G. Cahill, F.Hoffmann‐La Roche, Basle, Switzerland, internal memo dated 19 September 2008, unpublished Roche data). The removal was between 74 and 95% in 6 of these weeks, whereas in the 2 remaining wk there was no significant removal in one sample, but negative removal of –73% in the other. The memo does not give any information as to whether in the latter 2 wk, asynchronous sampling of influent and effluent may have led to no or negative removal, and the site has stopped production in the meantime. Still, including all 8 measurements, the average removal was 52% and the median removal was 83%, which clearly supports efficient biodegradation of MPM and MPA in activated sludge STPs. Therefore, the 3 SimpleTreat removal predictions in the previous paragraph were used for low, middle, and high removal scenarios and derivation of the corresponding refined PECs.

### Use data

Use data for MPM and MPA for the years 2004 to 2017 were retrieved from the IQVIA (2018) MIDAS database for the following 24 European countries: Austria, Belgium, Bulgaria, Croatia, the Czech Republic, Denmark, Finland, France, Germany, Greece, Ireland, Italy, Latvia, Luxembourg, The Netherlands, Poland, Portugal, Romania, Slovakia, Slovenia, Spain, Sweden, Switzerland, and the United Kingdom. For Greece, Latvia, and Luxembourg, only retail data were available (i.e., no hospital use data); however, this is not regarded as a major drawback, because MPM and MPA have to be taken on a daily basis over an indefinite time and therefore most of the use amounts will be at home, not in hospitals. This assumption is also supported by Franquet‐Griell et al. (2015, p 163). The 24 countries are estimated to comprise 504.7 million inhabitants in 2017, corresponding to approximately 93.4% of the total western and middle European population of 540.3 million (European Commission 2018). Therefore, the available data for MPA are regarded as representative for Europe.

### PECs and MECs

Because the single sales amounts are the intellectual property of IQVIA, the original data may not be shared as such, but only in processed form. The initial, overall‐use–based European MPA PECs and the refined average and single‐country highest‐use–based MPA PECs for STPs and receiving freshwaters were calculated as described in the *Materials and Methods* section. The refined PECs based on 3 different SimpleTreat removal predictions and ePiE PECs for the Rhine and Guadalquivir Rivers assuming only 12% STP removal are shown in Table 2. For the refined, country‐specific, water‐use‐ and dilution‐factor–based PECs, note that the highest PECs for STPs and receiving waters do not necessarily denote the same country, because low average water use will result in a high STP PEC but in case of subsequent high dilution this may still lead to a low surface water PEC. For the initial– and refined‐use–based PECs, the STP PECs ranged from 0.898 to 8.830 µg MPA/L, and the surface water PECs ranged from <0.001 to 0.532 µg MPA/L. The median refined European, population‐, water‐use‐, and dilution‐factor–weighted surface water PECs for 12, 43.6, and 73.1% STP removal were 0.058, 0.042, and 0.027 µg MPA/L, respectively. The ePiE distributions were calculated using all stretches in the respective ePiE catchments, that is, they included all those parts of rivers and tributaries upstream of the uppermost STP, where 0 concentrations are predicted. Under annual mean monthly flow conditions, the ePiE surface water PECs for the 4 catchments ranged from <0.001 to 5.495 µg MPA/L. This maximum corresponds to the 99th %ile of the Guadalquivir PECs, whereas the median Rhine and Guadalquivir ePiE PECs were 0.007 and <0.001 µg MPA/L. Under annual minimum monthly flow conditions, the worst‐case ePiE PECs were higher, with median PECs of 0.021 and <0.001 µg/L for the Rhine and Guadalquivir, and 99th %ile PECs of 2.666 and 12.419 µg/L, respectively. The extreme low‐flow ePiE PEC for the Guadalquivir is due to a high seasonal variability in river flow conditions. The ePiE mean‐flow surface water PECs for the Turia and Ouse catchments (Table 2) ranged from <0.001 to 0.318 and <0.001 to 0.534 µg MPA/L, respectively, with the median in both cases being <0.001 µg/L.

**Table 2:** Use‐based initial and refined sewage treatment plant and surface freshwater European predicted environmental concentrations (PECs) and measured environmental concentrations (MECs) for mycophenolic acid (MPA)

	Environmental compartment						
	STP PEC or MEC		Surface freshwaters PEC or MEC, ng/L, based on STP removal of				
PECs or MECs	µg/L	Location	0%	12%	43.6%	73.1%	NA
Average initial EMA EU PEC	2.990	Influent	0.299				
Average refined PEC per country	2.980	Influent		0.082	0.058	0.037	
Median refined PEC per country	2.960	Influent		0.058	0.042	0.027	
Highest refined PEC per country	8.830	Influent		0.532	0.383	0.245	
Lowest refined PEC per country	0.898	Influent		0.002^b^	0.001	<0.001	
Rhine catchment mean flow median ePiE PEC				0.007^b^			
Rhine catchment mean flow 99th percentile ePiE PEC				0.671^b^			
Rhine catchment mean flow 1st percentile ePiE PEC				<0.001^b^			
Rhine catchment low flow median ePiE PEC				0.021^b^			
Rhine catchment low flow 99th percentile ePiE PEC				2.666^b^			
Ouse catchment mean flow median ePiE PEC				0.048^b^			
Ouse catchment mean flow 99th percentile ePiE PEC				0.711^b^			
Ouse catchment mean flow 1st percentile ePiE PEC				0.001^b^			
Ouse catchment low flow median ePiE PEC				0.195^b^			
Ouse catchment low flow 99th percentile ePiE PEC				2.881^b^			
Turia catchment mean flow median ePiE PEC				0.028^b^			
Turia catchment mean flow 99th percentile ePiE PEC				0.554^b^			
Turia catchment mean flow 1st percentile ePiE PEC				<0.001^b^			
Turia catchment low flow median ePiE PEC				0.174^b^			
Turia catchment low flow 99th percentile ePiE PEC				4.959^b^			
Guadalquivir catchment mean flow median ePiE PEC				<0.001^b^			
Guadalquivir catchment mean flow 99th percentile ePiE PEC				5.495^b^			
Guadalquivir catchment mean flow 1st percentile ePiE PEC				<0.001^b^			
Guadalquivir catchment low flow median ePiE PEC				<0.001^b^			
Guadalquivir catchment low flow 99th percentile ePiE PEC				12.419^b^			
Maximum MEC	4.190^a^	Effluent					0.656^c^
Realistic worst‐case (90th percentile) MEC	ND						0.057^d^
Median MEC	~0.250^a^	Effluent					~0.002^d^

^a^ Highest and median of 6 available STP effluent MECs from Switzerland (Rossi and Cheseaux 2013).

^b^ Highest surface water MEC from River Besòs, Catalunya, Spain (Franquet‐Griell et al. 2016, 2017c).

^c^ 90th percentile and median of 110 available surface water MECs from Switzerland, Spain, and Poland (based on Rossi and Cheseaux 2013; Franquet‐riell et al. 2016, 2017c; Giebułtowicz and Nałęcz‐Jawecki 2016).

EMA EU PEC = MPA PEC based on overall European amounts of MPA and mycophenolate mofetil (MPM), no human metabolism beyond hydrolysis of MPM to MPA and no STP removal included, default dilution factor of 10 (Committee for Medicinal Products for Human Use 2015); ePiE PEC = geographical information system–based surface water PEC according to Oldenkamp et al. (2018); MEC = measured environmental concentration; PEC = predicted environmental concentration; MPA = mycophenolic acid; MPM = mycophenolate mofetil; NA = no data available; ND = not determined due to low number of MECs; STP = sewage treatment plant.

Franquet‐Griell et al. (2015) calculated an average STP effluent PEC of 2.008 µg MPA/L and a surface water PEC of 0.0774 µg MPA/L, based on the total MPA use in Catalunya over the last 3 yr before their publication, 41% removal in STPs, and the country‐specific dilution factor for Spain from Keller et al. (2014). In view of the uncertainties included (e.g., modeled STP removal and the dilution factor, which encompasses extremely dry regions in central and southern Spain), both of these PECs correspond reasonably well with the ones derived in the present study.

We only located a few surface water MECs for MPA, all dating from recent years. In 2013, Rossi and Cheseaux reported the presence of MPA in 3/3 samplings from the Swiss Rhone River upstream of Lake Geneva, at 0.0014, 0.0015, and 0.0021 µg/L, with a limit of quantitation (LOQ) of 0.001 µg/L and high uncertainty due to the low number of samples. Still, the 3 MECs, with an average of 0.0017 µg/L, correspond reasonably well with the refined use–based PEC for the whole of Switzerland assuming 73.1% STP removal of approximately 0.005 µg/L (data not shown).

In 2016, Franquet‐Griell et al. (2016) published 9/9 MPA surface water MECs for Spain, ranging from 0.0128 to 0.0562 µg/L (mean: 0.0217 µg/L), in the lower, heavily industrialized and urbanized River Llobregat in the south of Barcelona with a total of nearly 5 million people, and followed it through drinking water treatment to the finished water, where it was no longer detected (0/2; LOQ of 0.0001 µg/L; Franquet‐Griell et al. 2016). In the River Besòs in Catalunya, Spain, which runs through “a heavily populated and industrialized area, receiving the authorized discharges of 27 [STPs], 219 industries and 12 hospitals” in northern Barcelona, Franquet‐Griell et al. (2017c) measured cytostatic APIs. (Note that MPA is cytostatic only insofar as cell growth that depends on purine biosynthesis without salvaging pathways is inhibited, but not a cytostatic in the sense of classical cell division inhibitors, even though it is included in Franquet‐Griell et al. 2017c.) In 2 sampling campaigns in 2014, MPA was the target compound detected at the highest levels and was present mainly in the lowest, most urbanized river stretches, in 7/19 samples at 0.0131 to 0.0895 µg/L (mean: 0.018 µg/L, with nondetects counted as half the LOQ) in May and in 4/19 samples at 0.0085 to 0.656 µg/L (mean: 0.0474 µg/L, with nondetects counted as half LOQ) in July, with the latter, higher concentrations attributed by the authors to low water flow in summer. The dilution factor of the River Besòs is given as 1.2, showing very low dilution and a high fraction of treated wastewater (Franquet‐Griell et al. 2017c). The median combined MEC in Catalunya is just below 0.001 µg/L in view of many nondetects in the upper River Besòs, which is far lower than the refined use–based PEC for the whole of Spain assuming 73.1% STP removal of approximately 0.040 µg/L and lower again by another factor of 2 than the surface water PEC of 0.0774 µg MPA/L calculated by Franquet‐Griell et al. (2015) themselves. The median MEC of just below 0.001 µg/L is close to the median mean‐flow ePiE PECs for the Turia and Guadalquivir Rivers (both <0.001 µg/L), even though these river catchments are situated in different parts of Spain.

Giebułtowicz and Nałęcz‐Jawecki (2016) measured MPA in 2 rivers in Poland, the smaller Utrata and the large Vistula, in both cases upstream and downstream of STPs, in the Vistula downstream of the capital Warsaw, over 1 yr. They detected MPA 38/60 times in river water, from below an LOQ of 0.0005 to 0.180 µg/L downstream of a large STP, and 0/9 times in finished drinking water from the city of Warsaw. The median of Giebułtowicz and Nałęcz‐Jawecki's (2016) surface water MECs, including the nondetects, was close to 0.003 µg/L (mean: 0.0168 µg/L, with nondetects counted as half LOQ), which is more than a factor of 10 lower than the refined use–based PEC for Poland of just above 0.040 µg/L, assuming 73.1% STP removal.

A total of 110 published surface waters MECs for MPA have been located to date, with 61 above the LOQ. These values, including the nondetects, were compiled, back‐distributed, and percentage‐ranked according to Straub (2008), resulting in a compound median MEC of just below 0.002 µg/L (75th %ile ~0.017 µg/L, 90th %ile ~0.057 µg/L, 95th %ile ~0.123 µg/L, 99th %ile ~0.604 µg/L) and a maximum MEC of 0.656 µg MPA/L. The overall mean, with nondetects counted as half the LOQ, was 0.022 µg/L. In view of the low number of MECs and the restriction to Switzerland, Poland, and northeastern Spain, it is unknown whether these MECs and their distribution are representative for Europe. The single comparisons with refined PECs per country given in the previous paragraph consistently suggest an overestimation of the PECs by a factor of 3 for Switzerland, 13 for Poland, and 40 to 80 for Catalunya. The median MEC of <0.002 µg/L for the Rhone River is reasonably close, however, to the median mean‐flow Rhine River ePiE PEC of 0.007 µg/L. The overall distribution of MECs in comparison with the refined and ePiE PECs just described seems to support the notion of a too conservative PEC derivation. However, the low total and possibly skewed distribution of MECs must be kept in mind before accepting such conclusions as definitive.

Singer et al. (2016) identified and measured MPA in the effluent of 6 Swiss STPs, with an LOQ of 0.030 µg/L. They detected MPA in all 6 effluents with MECs of 0.065 to 4.190 µg/L (median: 0.755 µg/L). Based on only 6 MECs, the range and median compare reasonably well with the refined use‐ and water‐use–based STP PECs just described of 0.898 to 8.830 (median: 2.960) µg MPA/L.

Giebułtowicz and Nałęcz‐Jawecki (2016) also detected MPA “in very low concentrations [close to the limit of detection of 0.0015 µg/L] in sediment samples collected close to STPs. However, [... they] concluded that the detected MPA is freely dissolved in sediment porewater, which constituted approximately 30% of sediment samples prior to freeze drying” (Giebułtowicz and Nałęcz‐Jawecki 2016, p 144). This likely nonadsorption to sediment is in agreement with the new OECD test guideline 106 (Organisation for Economic Co‐operation and Development 2000; V. Halász‐Laky, Toxi‐Coop ZRT, Balatonfüred, Hungary, on behalf of F.Hoffmann‐La Roche, Basle, Switzerland, unpublished data).

Drinking water treatment removes MPA to untraceable concentrations (Franquet‐Griell et al. 2016; Giebułtowicz and Nałęcz‐Jawecki 2016). Giebułtowicz and Nałęcz‐Jawecki (2016) did not detect MPA in tap water in 9/9 samples in Poland. Similarly, Franquet‐Griell et al. (2016) showed that MPA from Llobregat River source water disappeared early in the drinking water treatment process and was not detected in 2/2 finished drinking water samples in Catalunya, Spain. Both groups had LOQs of 0.001 µg/L for MPA.

### Ecotoxicity

Syntex had MPA tested for acute daphnid ecotoxicity in compliance with GLP according to US Food and Drug Administration guideline 4.08 (2006), a test that resulted in a 48‐h median effect concentration (EC50) of 755 mg/L average measured concentration (AMC) and a 48‐h NOEC of 440 mg/L AMC (J.W. Blasberg and J. Bucksath, ABC Laboratories, Columbia MO, USA, on behalf of Syntex, Palo Alto, CA, USA/F.Hoffmann‐La Roche, Basle, Switzerland, unpublished data.). The public European Union European Chemicals Agency (2018) database gives a robust study summary for a static algal growth inhibition test according to OECD test guideline 201 (Organisation for Economic Co‐operation and Development 2011) in compliance with GLP with MPA in green algae of the species *Rhaphidocelis* (*Pseudokirchneriella*) *subcapitata.* The algae were exposed to MPA concentrations first for 24 h in the dark, and then for 72 h under continuous illumination. Analytical determinations of MPA concentrations were performed at 0, 24, and 96 h. Due to decrease in MPA concentrations over time, the geometric mean measured concentrations (GMMCs) for the 24‐ and 96‐h determinations were calculated. The ErC50 for MPA was 68 µg/L, the ErC10 was 12 µg/L, and the NOErC was 9 µg/L (all GMMC; European Chemicals Agency 2018). The Swedish FASS (2018) database gives the following ecotoxicity information (endpoints only) from Novartis for sodium‐MPA: the same algal data as above in the Registration, Evaluation, Authorisation and Restriction of Chemicals regulation, acute daphnid EC50 >100 mg/L in *D. magna* (OECD test guideline 202; Organisation for Economic Co‐operation and Development 1984) and acute fish 50% lethal concentration (LC50) in *Cyprinus carpio* of >100 mg/L (OECD test guideline 203; Organisation for Economic Co‐operation and Development 1993); also, an activated sludge respiration inhibition test according to OECD test guideline 209 (Organisation for Economic Co‐operation and Development 2010) resulted in a 3‐h EC50 of 2213 mg/L and an EC10 of 69 mg/L (FASS 2018). Because the latter 3 tests are from the same contract laboratory as the algal test above, they were probably performed in compliance with GLP as well. In addition, there is one older non‐GLP literature 16‐h LC50 value for the hypersaline water brine shrimp *Artemia salina* of 98.4 mg MPA/L (Ďuračková et al. 1977).

For terrestrial plant ecotoxicity, Wright (1951) examined the phytotoxic effects of some antibiotics on germination and root growth of wheat (*Triticum* sp.), white mustard *(Sinapis alba*), and red clover *(Trifolium pratense)* on agar containing different concentrations of test substances. The germination of mustard and clover seeds, but not wheat seeds, was significantly inhibited by MPA in a concentration‐dependent manner. However, MPA inhibited the root growth of all 3 plants with increasing concentration. Although no statistics are given in the publication, a concentration range of 1 to 5 ppm (1–5 mg/kg agar) emerges as the lowest‐observed‐effect concentration (LOEC) for both endpoints.

The new chronic ecotoxicity tests with cyanobacteria (D. Gilberg and G. Chambers, ECT Oekotoxikologie, Flörsheim, Germany, on behalf of F.Hoffmann‐La Roche, Basle, Switzerland, unpublished data), daphnids (P. Egeler and J. Chambers, ECT Oekotoxikologie, Flörsheim, Germany, on behalf of F.Hoffmann‐La Roche, Basle, Switzerland, unpublished data), and fish (D. Gilberg and G. Chambers, unpublished data) fulfilled the validity criteria of the respective OECD guidelines (see the Supplemental Data). In the new OECD test guideline 201 (Organisation for Economic Co‐operation and Development 2011), exposure of the cyanobacterium *A. flos‐aquae* resulted in a clear concentration–response relationship for both biological parameters growth rate and yield during the exposure period. The following endpoints were determined, all relating to GMMC: NOEC (both growth and yield) 83.9 µg MPA/L, LOEC 284 µg/L, ErC10 155 (95% confidence interval [CI] 137–171) µg/L, and ErC50 423 (95% CI 406–441) µg/L (D. Gilberg and G. Chambers, unpublished data).

In the new OECD test guideline 211 (Organisation for Economic Co‐operation and Development 2012a) reproduction test with *D. magna*, a clear dose–response relationship was found for fecundity and reproduction, with a NOEC of 630 µg MPA/L, a LOEC of 1800 µg/L, and an EC10 of 929 µg/L (no 95% CI possible), all GMMC. No clear dose–response relationship was found for the endpoint length of parental daphnids (NOEC 630 µg/L, LOEC 1800 µg/L, both GMMC), nor for the 2 endpoints mortality/immobility of parental daphnids and intrinsic rate of population increase, which both showed a NOEC at the highest tested concentration of 7530 µg MPA/L GMMC and a LOEC of >7530 µg/L (D. Gilberg and G. Chambers, unpublished data). Thus, the overall NOEC for the daphnids is 630 µg MPA/L and the overall EC10 is 929 µg/L, both GMMC.

In the fish partial life cycle test test with *D. rerio,* at the end of the OECD test guideline 229 (Organisation for Economic Co‐operation and Development (2012b) test phase at 23 d, no concentration–response relationship was observed for the parameters survival of the parental fish, fecundity, and fertilization success for the F0 generation. The NOEC of all endpoints was the highest tested nominal concentration of 100 µg MPA/L, corresponding to 73 µg/L GMMC. The EC10 for survival of the parental fish was 18.9 µg/L GMMC, whereas the EC10 for fecundity and fertilization success was calculated to be >73 µg MPA/L GMMC (D. Gilberg and G. Chambers, unpublished data). At the end of the OECD test guideline 210 (Organisation for Economic Co‐operation and Development 2013) test phase at 34 d (total duration of the test 57 d), no concentration–response relationship was observed for the parameter hatching success (NOEC 73 µg/L, EC10 >73 µg GMMC MPA/L), but a clear concentration–response relationship was observed for posthatch success (survival) and number of healthy fish, with a NOEC of 10.9 µg MPA/L and an EC10 of 5.8 µg MPA/L (GMMC; no CI), as well as length of the surviving F1 fish, with a NOEC of 1.32 µg/L and an EC10 of 31.9 (CI = 1.3–78) µg MPA/L, and finally wet weight of the surviving F1 fish with a NOEC of 1.32 µg/L and an EC10 of 10.6 (CI = 8–14.8) µg MPA/L (D. Gilberg and G. Chambers, unpublished data). At the highest tested concentration of 0.1 mg MPA/L nominal concentration (73 µg/L GMMC), only 2 of 75 hatched fish survived until the end of the test, showing that the test concentration range was well chosen. The most sensitive endpoints in this partial life cycle test were survival and growth in terms of length and mass of the young F1 fish; the effects were comparatively small but statistically significant at *p* = 0.05. In contrast, the F0 generation was impacted to a lesser extent. Thus, the overall NOEC for all endpoints in both F0 and F1 generations was 1.32 µg MPA/L, and the lowest EC10 was 5.8 µg MPA/L, both GMMC.

These relatively low (sub)chronic endpoints in fish are supported by Wu et al. (2006), who described a significant inhibition of angiogenesis of the intersegmental blood vessels of embryonic *D. rerio* exposed to MPA concentrations of ≥0.9 µmol/L (288 µg/L) in the short time window of 32 to 48 h post fertilization (hpf). In view of the rapid embryogenesis of *D. rerio,* Gao et al. (2014) exposed dechorionated zebrafish eggs from 2 to 72 hpf (when morphogenesis is basically complete) to different concentrations of MPA. As endpoints, heart rate and rhythm were assessed at 52 hpf, and pericardial edema, circulation, hemorrhage, and thrombosis were observed at 72 hpf. The MPA exposure resulted in pericardial edema at 6.92 µmol/L (2217 µg/L) and in abnormal body shape, axis shortening, enlarged yolk sac, and decreased motility at the lowest tested concentration of 1.38 µmol/L (442 µg/L); with the concentrations tested in that study, no NOEC was found. Thus, both Wu et al. (2006) and Gao et al. (2014) support developmental toxicity of MPA at concentrations <300 µg/L in short‐term embryotoxicity studies, in agreement with the partial life cycle test reported in the present study. In addition, Gao et al. (2014) also exposed *D. rerio* embryos at 72 hpf in an acute toxicity assay over 24 h; this test resulted in an LC50 of 55.4 µmol MPA/L (17.75 mg/L), which is clearly lower than the results reported for juvenile carp over 96 h in an OECD test guideline 203 (Organisation for Economic Co‐operation and Development 1993) test of >100 mg/L (European Chemicals Agency 2018) and may evidence increased sensitivity of embryonic stages.

On a broader scale of comparison, developmental toxicity as observed in *D. rerio* was also observed in Wistar rat embryos, murine embryonic stem cells, and mouse fibroblasts exposed to MPA (Eckardt and Stahlmann 2010), and in chicken embryos (Veselý and Veselá 1991, publication in Czech language, seen only as the abstract). Thus MPA has been seen to cause proliferation and cellular and developmental toxicity to mammalian and avian embryos, stem cells, or fibroblasts at concentrations of ≥31 µg/L in mammalian systems, in agreement with the effects seen in fish.

### Sewage treatment and aquatic PNECs

The microorganism PNEC for STPs was derived from the activated sludge respiration inhibition test according to OECD test guideline 209 (Organisation for Economic Co‐operation and Development 2010), which resulted in a 3‐h EC50 of 2213 mg/L and an EC10 of 69 mg/L (European Chemicals Agency 2018), by applying an assessment factor of 10 to the EC10, resulting in a PNEC_STP_ of 6900 µg MPA/L. This finding is indirectly supported by the only tested concentration of 20 µg MPA/L in the OECD test guideline 314B (Organisation for Economic Co‐operation and Development 2008) biodegradation study, in which biodegradation began without any delay or lag phase (T. Junker and M. Herrchen, unpublished data), and also by a ready biodegradability study with MPM, which showed no inhibition of activated sludge activity at a nominal concentration of 100 mg MPM/L (A. Häner, unpublished data).

The NOEC‐based aquatic PNEC (PNEC_NOEC_) was derived from the lowest chronic NOEC from the tests with green algae, cyanobacteria, daphnids, and fish with an assessment factor of 10, resulting in a PNEC_NOEC_ of 0.132 µg MPA/L based on the fish NOEC. The EC10‐based PNEC_EC10_ was derived from the same tests, resulting in 0.58 µg MPA/L, also based on fish, which were the most sensitive species among the 4 groups tested.

### Aquatic environmental risk assessment

For STPs, PECs or MECs were compared with the PNEC STP by calculating the risk quotient PEC/PNEC. The highest refined PEC_STP_ was 8.83 µg/L (Table 2), and the highest STP effluent MEC was 4.19 µg/L (Singer et al. 2016); the PNEC_STP_ based on OECD test guideline 209 (Organisation for Economic Co‐operation and Development 2010; FASS 2018) was 6900 µg/L. Thus, the highest PEC‐based risk quotient for STPs was 0.00128, and the highest MEC‐based risk quotient was 0.0006. These low risk quotients suggest no significant risk for STPs from the use of MPM and MPA.

**Figure 3:**
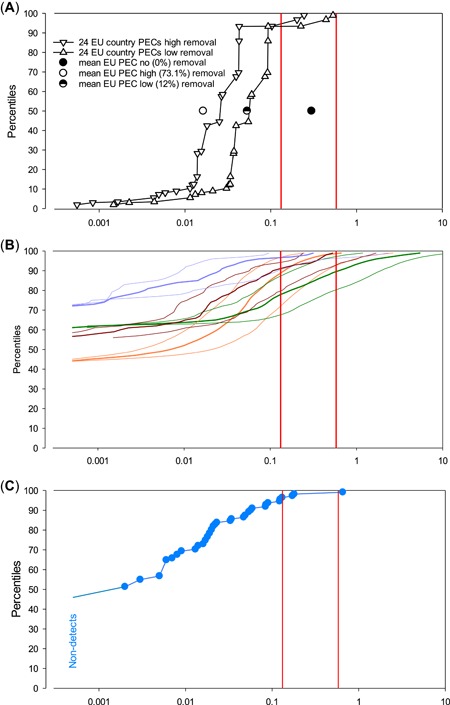
Risk graphs based on predicted environmental concentration (PEC) and measured environmental concentration (MEC) for mycophenolic acid (MPA). (**A**) PECs. Filled black circle = use‐based initial European Union PEC without removal in sewage treatment plants (= initial European Medicines Agency PEC); half‐filled circle = European Union PEC with 12% removal; open circle = European Union PEC with 73.1% removal. (**B**) Exposure to pharmaceuticals in the environment (ePiE) PEC distributions, assuming 12% removal in sewage treatment plants. Fat mauve line = Turia catchment mean‐flow PEC; fine mauve lines = Turia catchment high‐flow (to left of mean) and low‐flow (to right of mean) PECs. Same for brown lines = Ouse catchment; orange lines = Rhine catchment; green lines = Guadalquivir catchment. The PECs were cut off at <0.0005 µg/L. (**C**) MECs. Percent‐ranked distribution of 110 MPA MECs from Switzerland, Spain, and Poland; nondetects (limit of quantitation = 0.001 µg/L) increase the rank of the lowest shown detected MEC. In all panels, no‐observed‐effect concentration (NOEC)‐derived (0.132 µg/L, left) and 10% effect concentration (EC10)‐derived (0.580 µg/L, right) predicted no‐effect concentrations (PNECs) are shown as vertical red lines. Symbols to the left of the PNEC_NOEC_ or PNEC_EC10_ show no significant risk for the respective PNEC, and symbols to the right show potential risk. EU = European Union.

Similarly, for receiving freshwaters, the initial EMA PEC was 0.299 µg/L, and the average and median refined PECs, depending on STP removal, were quite close together, with 0.082 and 0.058 µg/L for 12% removal, 0.058 and 0.042 µg/L for 43.6% removal, and 0.037 and 0.027 µg/L for 73.1% removal; the highest refined PECs per country were 0.532, 0.383, and 0.245 µg/L for the 3 removal rates, respectively. The aquatic PNEC_NOEC_ was 0.132 µg/L, and the PNEC_EC10_ was 0.580 µg/L. The corresponding risk quotients for the PNEC_EC10_ are shown in Table 3 (showing the PNEC_NOEC_ risk quotient as well would inflate the table even more). Some cases with a risk quotient >1 suggest there may be risk to surface waters. The situation becomes even more complex with a distribution of all MECs, of the population‐weighted refined use‐based PECs for the 24 countries and of the ePiE PECs, the latter including both %ile distributions and mean‐, low‐, and high‐flow conditions. Therefore, these risks are shown graphically in Figure 3.

**Table 3:** Surface water risk quotients for predicted no‐effect concentration based on 10% effect concentration (PNEC_EC10_; 0.58 µg mycophenolic acid [MPA]/L)

	Surface freshwaters risk quotients, based on STP removal of				
PECs or MECs	0%	12%	43.6%	73.1%	NA
Average initial EMA EU PEC	0.516				
Average refined PEC per country		0.141	0.100	0.064	
Median refined PEC per country		0.100	0.072	0.047	
Highest refined PEC per country		0.917	0.660	0.422	
Lowest refined PEC per country		0.003	0.002	<0.002	
Rhine catchment mean flow median ePiE PEC		0.102			
Rhine catchment mean flow 99th percentile ePiE PEC		1.157			
Rhine catchment mean flow 1st percentile ePiE PEC		<0.002			
Rhine catchment low flow median ePiE PEC		0.036			
Rhine catchment low flow 99th percentile ePiE PEC		4.597			
Ouse catchment mean flow median ePiE PEC		0.083			
Ouse catchment mean flow 99th percentile ePiE PEC		1.226			
Ouse catchment mean flow 1st percentile ePiE PEC		0.002			
Ouse catchment low flow median ePiE PEC		0.336			
Ouse catchment low flow 99th percentile ePiE PEC		4.967			
Turia catchment mean flow median ePiE PEC		0.048			
Turia catchment mean flow 99th percentile ePiE PEC		0.955			
Turia catchment mean flow 1st percentile ePiE PEC		<0.002			
Turia catchment low flow median ePiE PEC		0.300			
Turia catchment low flow 99th percentile ePiE PEC		7.922			
Guadalquivir catchment mean flow median ePiE PEC		<0.002			
Guadalquivir catchment mean flow 99th percentile ePiE PEC		9.474			
Guadalquivir catchment mean flow 1st percentile ePiE PEC		<0.002			
Guadalquivir catchment low flow median ePiE PEC		<0.002			
Guadalquivir catchment low flow 99th percentile ePiE PEC		21.41			
Maximum MEC					1.131
Realistic worst‐case (90th percentile) MEC					0.983
Median MEC					~0.003

EMA EU PEC = MPA PEC based on overall European amounts of MPA and mycophenolate mofetil (MPM), no human metabolism beyond hydrolysis of MPM to MPA and no STP removal included, default dilution factor of 10 (European Medicines Agency 2015); ePiE PEC = geographical information system–based surface water PEC according to Oldenkamp et al. (2018); MEC = maximum, 90th percentile and median of 110 available surface water measured environmental concentrations (MECs) from Switzerland, Spain, and Poland (based on Rossi and Cheseaux 2013; Franquet‐Griell et al. 2016, 2017c; Giebułtowicz and Nałęcz‐Jawecki 2016); PEC = predicted environmental concentration; MPM = mycophenolate mofetil; STP = sewage treatment plant; NA = no data available.

All PECs or MECs with a risk quotient >1 (i.e., >0.132 µg MPA/L for PNEC_NOEC_ or >0.580 µg MPA/L for PNEC_EC10_) signify potential risk. The initial use‐based Europe‐wide PEC (black filled circle in Figure 3A) shows a risk quotient of 2.27 for the PNEC_NOEC_ but no significant risk (risk quotient 0.516) for the PNEC_EC10_. The percent‐ranked refined, total‐MPA‐use‐, water‐use‐, and dilution‐factor–based PECs per country assuming 12% (black triangles pointing down) or 73.1% (black triangles pointing up) removal in STPs showed no significant risk based on the PNEC_NOEC_ for 21 of 24 countries, or >94% of instances referring to total population, with 3 countries (<6% of total population) potentially at risk. All the latter 3 countries are characterized by a combination of above average total MPA use and below average water use times receiving water dilution. The average and median refined PECs were 0.082 and 0.088 µg MPA/L, respectively, for low (12%) STP removal, and the average and median refined PECs were 0.037 and 0.027 µg MPA/L, respectively, for high (73.1%) STP removal. Hence, for both averages and medians, the PNEC_NOEC_ indicates no significant risk. Based on the PNEC_EC10_, none of the 24 countries in both low‐ and high‐removal scenarios show potential risk.

The mean‐flow ePiE PEC distribution for the Guadalquivir, Ouse, Rhine, and Turia catchments are shown in Figure 3B, as thick green, brown, orange, and mauve lines, with the low‐flow PECs in the same colors to the right and the high‐flow PECs to the left. All ePiE PEC lines have in common that the graphed values only start the PECs at ≥0.0005 µg/L, which corresponds to half the LOQ for the MECs; lower values are not shown. The mean‐flow ePiE PEC distribution for the Rhine catchment (orange lines) suggests no significant risk for approximately 89% based on the PNEC_NOEC_ (left vertical red line, Figure 3B) and, vice versa, potential risk for approximately 11% of the whole Rhine catchment; based on the PNEC_EC10_ (right vertical red line), no significant risk is indicated up to approximately 97.5% and a potential risk for approximately 2.5% of the whole catchment. Under low flow conditions, the percentages at no risk decreased to approximately 72 and approximately 92% based on the PNEC_NOEC_ and the PNEC_EC10_, respectively. The mean‐flow ePiE PEC distribution for the Guadalquivir catchment (green lines) suggests no significant risk for approximately 78% based on the PNEC_NOEC_ and >89% based on the PNEC_EC10_; for the worst‐case low‐flow Guadalquivir PECs, no significant risk appeared for approximately 68% based on the PNEC_NOEC_ and for approximately 80% based on the PNEC_EC10_. The lower inclination of the Guadalquivir PECs in comparison with the Rhine in Figure 3 is likely the result of a higher spatial variation in concentrations; this can be due to geographical variation in flow and/or population density (consider the presence of Cordoba and Seville in this midsize river basin), plus all the factors that influence concentrations in a warm Mediterranean climate zone. For the Turia catchment (mauve lines) mean flow, no risk emerged for 96.5% for PNEC_NOEC_ and >99% for PNEC_EC10_; for Turia low flow, there was no risk for 90 and 96%, respectively. Lastly, for the Ouse catchment (brown lines) mean flow, there was no significant risk for 91% for PNEC_NOEC_ and >99% for PNEC_EC10_; for Ouse low flow, there was no risk for 80 and 93%, respectively.

All available MECs were percentage ranked and graphed and are shown in Figure 3C. Of the total of 110 MECs (bright blue dots), 51 (46.4%) were below the LOQ. The remaining MECs increased up to a maximum of 0.656 µg MPA/L. Five of the 110 MECs (4.6%) were above the PNEC_NOEC_ of 0.132 µg/L, whereas only 1 (0.91%) was higher than the PNEC_EC10_ of 0.580 µg/L.

Figure 3 shows first that the distribution of the MECs groups nicely with the ePiE PECs: quantifiable (>0.001 µg/L) MECs start at approximately 50% of the total number of samples, which is not too far from that of the ePiE PECs. Also, the general inclination and highest MEC fit quite well into the ePiE PECs. However, this fit might be an artifact, seeing that the MECs are from Switzerland (specifically, a part that does not drain into the Rhine catchment), Poland (which is not yet incorporated into the ePiE), and northeastern Spain (which is not part of the Turia or Guadalquivir catchments and climatically not the same as southern Spain). Hence, the fit may represent a genuine property of use, STP removal, and environmental fate of MPA, but it might be coincidence, or somewhere in between. Also, the comparison of the refined PECs per country, in particular with high (73.1%) STP removal, may be tricky, because compound MECs from only 3 countries, with widely varying numbers of measurements, are being compared with a distribution of 24 single‐country average PECs. Still, reasonable commonalities are the upper (99th %ile) ends of all distributions, which range between 0.1 and nearly 10 µg/L and which are certainly dependent on the total amount of MPA introduced into all models and samples. Also, the European Union‐wide PECs without removal and with low and high removal in STPs agree quite well overall with the ePiE PECs and the MECs, but also with the single‐country PECs with low and high removal. Although it is recognized that the comparisons are not straightforward and may be equivocal, it is proposed that there is sufficient overlap to accept some commonalities, specifically in the upper regions of PECs and MECs, so that a comparison of the PEC and MEC distributions seems reasonably well founded.

### Antibiotic resistance risk assessment

Noto et al. (1969) tested the activity of MPA against various microorganisms, both fungi and some strains of bacteria. For 12 strains of *S. aureus* they determined MICs between 31.25 and 125 mg MPA/L; for *Staphylococcus epidermidis* and 2 strains each of *Shigella flexneri, Proteus vulgaris, Escherichia coli, Pseudomonas aeruginosa*, and *Salmonella enteritidis*, they found MICs of 125 to >500 mg MPA/L. These high MIC values characterize MPA as a rather weak antibiotic. Higher inhibition of MPA was shown against pathogenic fungi of the genera *Candida, Willia, Cryptococcus, Microsporum, Aspergillus*, and *Trichophyton*, with MICs ranging from 3.9 to 500 mg/L (Noto et al. 1969). Based on the 23 bacterial MICs given by Noto et al. (1969), a provisional PNEC_ABR_ for MPA can be derived following Kümmerer and Henninger (2003) by dividing the lowest MIC by 100, resulting in a first PNEC_ABR_ of 312.5 µg/L. The procedure described by Bengtsson‐Palme and Larsson (2016) results in a second provisional PNEC_ABR_ for MPA, calculated by J. Bengtsson‐Palme, of “76 µg/L (which would be rounded down to 64 µg/L”; J. Bengtsson‐Palme, personal communication to J.O. Straub, 25 July 2018). Selecting the lower of the 2 values results in a provisional PNEC_ABR_ of 64 µg MPA/L, based on a small MIC dataset.

This provisional PNEC_ABR_ of 64 µg MPA/L can now be compared with the PECs for STPs and surface water and with the highest MECs available. The highest use‐based refined PEC_STP_ was 8.83 µg MPA/L (Table 2), whereas the highest STP effluent MEC was 4.19 µg/L (Singer et al. 2016); the corresponding risk quotients were 0.138 and 0.065, respectively, suggesting no significant antibiotic resistance risk for STPs. For surface waters, all PECs including the highest, the Guadalquivir River low‐flow 99th %ile ePiE PEC, were below the PNEC_ABR_, and thus all risk quotients were ≤0.194, all refined PEC‐per‐country risk quotients were ≤0.0083, and all mean‐ and low‐flow median ePiE PEC risk quotients were ≤0.00033; finally, the highest MEC risk quotient was 0.0103. Both models and measurements imply that throughout Europe, even in the worst‐case scenario of a southern Mediterranean catchment with extremely low river flow in summer, no significant antibiotic resistance risk emerges for MPA.

### Secondary poisoning for (semi)aquatic top predators and humans

Risk assessments for secondary poisoning of both (semi)aquatic top predators and human consumers of water and fish depend on mammalian toxicity data. Co‐author T. Pfister collated and assessed such data for the derivation of an ADE value for MPA (T. Pfister, unpublished data). According to these same data, based on chronic and subchronic studies with mice, rats, dogs, and monkeys, adverse effects on the hematopoietic and lymphoid system were identified as the leading changes; the rat was the most sensitive species, and the no‐observed‐adverse‐effect level (NOAEL) after chronic (6 or 12 mo) oral treatment was established at 2 mg/kg body weight/d. The anticipated therapeutic effect, immunosuppression, was achieved at or below no‐effect dose levels for toxicity in (sub)chronic studies in rat and monkey, as assessed in vitro by the effect of serum from dosed animals. In terms of genotoxicity, MPM did not induce point mutations or primary DNA damage in the presence or absence of metabolic activation. More recent studies with extended exposure conditions have shown that MPM possesses a clastogenic potential, which becomes expressed only at highly cytotoxic dose levels, apparently as a consequence of purine synthesis inhibition. It was not carcinogenic in mice dosed orally for 104 wk at 25, 75, or 180 mg/kg body weight/d, or in rats dosed for at least 104 wk at 3, 7, or 15 mg/kg body weight/d. On the contrary, in secondary pharmacology studies (T. Pfister, unpublished data), MPM demonstrated in vitro antitumour effects in lymphocyte and erythrocyte systems, and MPM prolonged survival times in mice with large cell lymphoma. In a female fertility and reproduction study in rats dosed orally, the highest dose of 4.5 mg/kg body weight/d caused malformations (mainly of the head and eyes) in the pups in the absence of maternal toxicity, whereas there was no effect on fertility; the NOAEL was 1.5 mg/kg body weight/d. In teratology studies, rats and rabbits were dosed orally daily, resulting in deformities including head and ventral wall abnormalities in the rat and cardiovascular, kidney, and lung effects in the rabbit; the no‐observed‐effect level (NOEL) for teratogenic changes was 2 mg/kg body weight/d for rats and 30 mg/kg body weight/d for rabbits (T. Pfister, unpublished data).

The ADE was derived based on the available nonclinical and clinical data, with the critical effects identified as 1) immunosuppression and secondary effects thereof and 2) teratogenicity; in addition, the relevance of positive results in genotoxicity tests was assessed (T. Pfister, unpublished data). Based on the NOAEL of 2 mg/kg body weight/d in the 12‐mo oral toxicity study in the rat, an ADE was derived by multiplying this NOAEL by a default body weight of 60 kg for humans and dividing by adjustment factors, namely, 6.2 for extrapolation from rats to humans, 10 for variability between individuals, 1 for study duration of 1 yr for rodents, 5 for severity of systemic toxicity (hematopoiesis, immunotoxicity), and 3 for use of an established NOAEL instead of an NOEL; this derivation results in a first ADE of 0.1 mg MPM/60‐kg person/d. Based on the NOEL of 2 mg/kg body weight/d in the teratogenicity study in the rat, an ADE was derived by multiplying this NOEL by a default body weight of 60 kg for humans and dividing by adjustment factors, namely, 6.2 for extrapolation from rats to humans, 10 for variability between individuals, 1 for reproductive studies in which the whole period of organogenesis was covered, 10 for a teratogenic effect without maternal toxicity, and 1 for use of an established NOEL; this derivation resulted in a second ADE of 0.19 mg MPM/60‐kg person/d (T. Pfister, unpublished data). The weak clastogenic potential observed at higher doses was considered to be irrelevant at these exposure levels. Because the first ADE was lower than, and therefore also protective of, the second, 0.1 mg MPM/60‐kg person/d was selected as the ADE. Stoichiometrically, this ADE for MPM corresponds to 0.0739 mg MPA/d, which was rounded to 0.075 mg or 75 µg MPA/d. The MTDI for an otter was derived by normalizing the ADE for a 60‐kg human to the otter with a default body mass of 10 kg, resulting in an MTDI of 0.0125 mg or 12.5 µg MPA/10‐kg otter/d.

For the top predator risk assessment through secondary poisoning, an otter is assumed to consume 1 kg of fish plus 0.79 L water every day (European Commission 2011; Murray‐Smith et al. 2012). The fish will take up MPA from the water with the highest modeled BCF, the geometric average of the SciFinder values in the pH range of 5 to 9, of 6.22 (see the previous section, *Physicochemical properties and environmental fate*). Using the Rhine River mean‐flow 99th %ile ePiE PEC of 0.671 µg/L, which is close to the highest MEC available of 0.656 µg/L, and the BCF of 6.22, results in 4.17 µg MPA/kg fish. In addition, the otter drinks 0.79 L of water at 0.671 µg MPA/L or 0.53 µg MPA/d. Thus, the total MPA uptake for an otter is 4.7 µg MPA/d. This is below the MTDI of 12.5 µg MPA/d, with a risk quotient of 0.376. Using the Rhine River low‐flow 99th %ile ePiE PEC, in contrast, results in a risk quotient of 43.2, suggesting potential risk. As worst‐case scenarios, the highest Guadalquivir River mean‐ and low‐flow 99th %ile ePiE PECs result in risk quotients of 88.9 and 201, respectively. These risk quotients suggest potential risk in extreme exposure scenarios to top predators like otters, which are reported to occur in the whole of Europe including the Iberian Peninsula and the Guadalquivir catchment (International Union for Conservation of Nature 2019). As for the aquatic risk assessment, the high ends of the ePiE PEC distributions signal potential risk, whereas the median refined and ePiE PECs suggest no significant risk.

For the human secondary poisoning risk assessment, a default 60‐kg person is expected to consume 2 L of drinking water/d (European Commission 2011; Murray‐Smith et al. 2012). Assuming as a worst case that no elimination of MPA takes place during drinking water treatment (despite the evidence to the contrary cited previously), this corresponds to 1.312 µg MPA/d. Using the data calculated just above for the otter, an amount of 115 g fish meat is predicted to contain 0.480 µg MPA. Therefore, a human is expected to consume a total of 1.792 µg MPA/d through secondary poisoning, which is well below the ADE of 75 µg/d, with a risk quotient of 0.024. For human secondary poisoning, even the extreme Guadalquivir River low‐flow 99th %ile ePiE PEC of 12.419 µg MPA/L does not result in a significant risk, with a risk quotient of 0.442. Hence, there is no significant risk for human secondary poisoning.

### Persistence, bioaccumulation, and toxicity assessment

The water/sediment test (Z. Yan, unpublished data), the OECD test guideline 314B (Organisation for Economic Co‐operation and Development 2008) biodegradation test (T. Junker and M. Herrchen, unpublished data), and the *k*_biodeg_ results from Franquet‐Griell et al. (2017a) suggest that MPA is not persistent. Furthermore, measured log *D*_OW_ values of 2.28 at pH 5, 0.48 at pH 7, and ≤–1.54 at pH 9 (V. Nicholson et al., unpublished data) suggest that MPA is not bioaccumulative, which is supported by modeled BCFs (US Environmental Protection Agency 2016; American Chemical Society 2018). Lastly, MPA is toxic based on mammalian carcinogenic, mutagenic, or reprotoxic properties (T. Pfister, unpublished data) and on chronic ecotoxicity in green algae and fish with NOECs <10 µg/L (European Chemicals Agency 2018; D. Gilberg and G. Chambers, unpublished data). Overall, even though it is toxic, MPA is not classified as persistent, bioaccumulative, or of specifically high ecotoxicity.

## DISCUSSION

Once taken up, MPA is mainly metabolized by conjugation to the inactive glucuronide MPAG and basically excreted without further metabolism, with a terminal half‐life of approximately 18 h; less than 1% of MPA is phase‐I‐metabolized to 6‐O‐desmethyl‐MPA (Wishart et al. 2018; Royal Pharmaceutical Society 2018). Glucuronides are cleft in STPs (Möhle and Metzger 2001), resulting in free MPA. It was shown in the OECD test guideline 314B (Organisation for Economic Co‐operation and Development 2008) test that [^14^C]‐MPA was biodegradable in aerobic activated sludge (T. Junker and M. Herrchen, unpublished data), confirming the finding by Franquet‐Griell et al. (2017a). The biodegradation reaction constant *k*_biodeg_ from the new OECD test guideline 314B (Organisation for Economic Co‐operation and Development 2008) test was entered into SimpleTreat 4.0, which calculates a removal of 12% for a standard STP with primary sludge settler, aeration basin, and a final settling tank, without nitrification/denitrification or further treatment. However, Franquet‐Griell et al. (2017a) showed in their sequencing batch reactor that the *k*_biodeg_ for MPA increases with every cycle of their reactor; their maximum *k*_biodeg_ in the fifth cycle results in a SimpleTreat removal of 73%. Because repeat exposure is certain for an API with daily administration over a lifetime, the PECs derived using 12% removal, including the ePiE PECs, constitute worst‐case assumptions.

These PECs and the few MECs are compared with PNECs derived from chronic effects in green algae, cyanobacteria, daphnids, and fish. *Daphnia* show the least sensitivity to MPA, whereas the blue—green *Anabaena* are approximately 8 times, green algae 70 times, and fish nearly 500 times more sensitive than daphnids. The strong effect on fish is primarily manifested in posthatch survival and growth parameters (length, body weight) of the fry, but not in survival of the parental generation, nor in fecundity, fertilization, hatchability, or obvious malformations (D. Gilberg and G. Chambers, unpublished data). The relatively nonspecific endpoints responsible for the NOEC and EC10 may indicate an increased general stress for the fish, potentially caused by the pharmacological mode of action, immune inhibition. The finding that cyanobacteria are less sensitive to MPA (D. Gilberg and G. Chambers, unpublished data) than green algae (European Chemicals Agency 2018) may first of all confirm that MPA is a rather weak antibiotic. The green algae were pre‐exposed to MPA in the dark for 24 h before the actual 72‐h testing period under illumination started, meaning that the total exposure duration was 96 h, in contrast to the cyanobacteria exposure, which totaled 72 h (European Chemicals Agency 2018). Also, as noted in the *Introduction*, IMDPH inhibition may be lower in prokaryotes than in eukaryotes (Digits and Hedstrom 1999; Gunnarsson et al., 2008), but this might be contradicted by the even lower sensitivity of the daphnids. Otherwise, the observed 9‐fold difference in sensitivity between green algae and cyanobacteria remains unexplained. Lastly, the daphnids had the least sensitivity to MPA, with the lowest NOEC of 630 µg/L GMMC for the endpoints reproduction/fecundity and length of parental daphnids, whereas for mortality/immobility of parental daphnids and intrinsic rate of population increase, the NOEC was 7530 µg/L GMMC, the highest tested concentration in this assay; the only EC10 that could be calculated was for the endpoint reproduction/fecundity, namely, 929 µg/L (Egeler and Chambers 2018). This finding suggests that parameters linked to overall fitness were impacted first (reproduction and fecundity plus length [size and growth] of the parental daphnids) before parameters evidencing overt toxicity (mortality/immobilization) and population growth (intrinsic rate) were affected. As for fish, this may indicate an increased general stress for the daphnids, potentially caused by the pharmacological mode of action. Immune inhibition due to MPA will be investigated, and its sensitivity compared with that of the standard chronic ecotoxicity endpoints for daphnids and fish, in a separate study.

The present environmental risk assessment derives PNECs from both chronic NOEC and EC10 values. Both are approximations of a true no‐effect concentration (NEC), which would ideally be used for PNEC derivation. Both NOEC and EC10 values have their biological or statistical advantages and shortcomings, depending on a clear concentration–response relationship and the shape and slope of the concentration–response curve, or on statistics applied, as discussed extensively (e.g., Kooijman 1981; van der Hoeven et al. 1997; Organisation for Economic Co‐operation and Development 2003), but both are in use. The NOEC has an additional uncertainty that depends on the spacing factor between the test concentrations (in addition to the actual biological effect), because the actual NEC may be precisely at, or nearly one spacing factor below, the determined NOEC. As a further shortcoming, the NOEC depends on just one concentration, even if data for higher test concentrations are available. In contrast, in a series with clear concentration–response effects, the EC10 integrates information from other test concentrations as well. Because there is a clear dose–response relationship with relatively steep curves in the ecotoxicity tests with MPA, the PNEC derivation from the EC10 values is given preference in the present study. Using the EC10 for the PNEC in human pharmaceutical environmental risk assessment is not only accepted but is actually preferred in the recent EMA (European Medicines Agency 2018) draft guideline. This does not mean, however, that an EC10 is always higher (i.e., a less sensitive endpoint) than a NOEC. Specifically for the abbreviated life cycle test with MPA reported in the present study, the lowest NOECs for single endpoints are 1.32 µg/L for both length and wet weight of the surviving F1 fish and 10.9 µg/L for posthatch survival and externally healthy fish; in contrast, the EC10 values are 31.9 µg/L for length and 10.6 µg/L for weight, but 5.8 µg/L for posthatch survival and health (D. Gilberg and G. Chambers, unpublished data).

The mean‐ and low‐flow 99th %ile ePiE PECs for the Rhine, Guadalquivir, Turia, and Ouse catchments, but also the 5 highest MECs retrieved, show potential risk for one or both of the PNECs. On the “safe” side, both the average and median refined PECs per country as well as the mean‐flow median and 75th %ile ePiE PECs for the Guadalquivir, Ouse, Rhine, and Turia catchments, but also the median and the 90th %ile of the surface water MECs show risk quotients of <1. The value of ePiE as a predictive instrument appears in these different catchments and scenarios. Instead of simplistic “one‐value” estimates, ePiE models different seasonal flow rates, different climate influences, different population densities, and different STP treatments. This results in complicated but, based on the limited comparison with MECs, quite reasonable distributions that can be used for worst‐ and average‐case assessment of risks (Table 3 and Figure 3C). In addition, ePiE can be used on a much finer spatial resolution than whole‐river catchments, which allows researchers to pinpoint hot spots, stretches, and regions that show higher concentrations and potential risk than others and thereby, for instance, to identify STPs that would profit most from upgrading. Deciding which STPs will be retrofitted is a highly political task and clearly beyond this risk assessment. However, solutions must be sought that do not just penalize the downstream STP that causes the combined PEC to exceed the PNEC while all upstream STPs (which contribute to the overall PEC) need not upgrade (or share in the costs) at all. A fair distribution of upgrading and financing even across state borders may become necessary.

Obviously, a risk characterization for MPA in European surface waters cannot be a simplistic “risk” or “no risk” conclusion, but will need differentiated discussion. Figure 3 is intended to support this evaluation by allowing one to derive percentage figures for potential risk versus no significant risk for the different PEC and MEC scenarios. Thus, the resulting aquatic environmental risk assessment for MPA in Europe describes a complex situation in which in the current deterministic risk scenarios some risk quotients are >1 (<6% of all refined PECs and MECs) whereas the majority by far (>94% of refined‐PEC–based and >95% of MEC‐based risk quotients) shows no significant risk. For the ePiE PEC distributions, in view of its inherently distributional concept, the environmental risk assessment is even more varied because it includes 4 different catchments with high, median, and low river flow scenarios, and the results are presented as a distribution from the 1st to the 99th %iles (Figure 3). Also, the comparable results for the aquatic top predators support the use of a differentiated environmental risk assessment. The following questions arise from these results: “What does’ significant risk’ or 'no significant risk’ mean?,” “Must a risk quotient <1 apply in all instances examined?,” or, vice versa, “What percentage of risk quotient >1 are we willing to accept?” These are partly scientific but also societal and political questions; a rational discussion with scientific input is clearly needed.

The concepts developed in probabilistic environmental risk assessment (e.g., Campbell et al. 1999; Hart 2001) could possibly help. Probabilistic environmental risk assessment recognizes that both (eco)toxicity and environmental exposure cannot be reduced to one single value each. Predictions (or measures) of both effects and exposures are just parts of distributions. Comparing these distributions elucidates where they overlap—this intersection outlines the area of potential risk. Also, the overlap allows a quantification, in terms of both share of species and share of environmental concentrations at risk (e.g., Straub 2008). Moreover, the goal of environmental risk assessment is generally not considered one that would protect every single individual organism, in contrast to human health risk assessment. For environmental risk assessment, the main objective is the protection of ecosystem functions and basic structure. This means that, within limits and with due consideration, a certain amount of risk may be tolerated as long as the ecosystem as a whole continues to function, for example, by organisms taking over the functions of other organisms, by replenishment of impaired organisms through immigration, by the development of resilience, or through a limited spatial extent of risk. The present environmental risk assessment, which is distributional (in the geographical sense) for the PECs and MECs, but deterministic for the ecotoxicity, shows quantifiable overlap on the exposure side. How much overlap or percentages at risk can be tolerated must be informed by science, mainly field ecology but also ecotoxicology. However, other considerations need to be integrated as well, for example*,* the usefulness and necessity of the API, existing alternatives, and the value that society accords to ecosystems and human health, but also the possibility and cost of risk reduction measures. Again, scientific, societal, and political questions must be addressed.

When these results are judged, it should be kept in mind that uncertainty is an inherent property of risk assessment (Wilson and Crouch 1987; Suter 1990; Darbra et al. 2008) and that such uncertainty applies to PECs, MECs, PNECs, and the resulting risk quotients. Although the refined and ePiE PEC distributions per country are comparable, this may primarily reflect a basic similarity in the procedures of generating PECs, in addition to (hopefully) a good measure of realism in the algorithms. Also, the MEC distributions may be strongly skewed, because the MEC data are scarce (only 3 countries [or regions] and a high variation in numbers of MECs/country), so the seemingly comparable picture for the MECs may or may not be an artifact. However, all distributions do have in common the result that the general distributions of ePiE PECs and the MECs are similar and, in particular, that some, a limited percentage, of the different PECs and MECs are higher than the PNECs. How much higher exactly depends on the data behind the distributions—a further uncertainty. Also, the PNECs, whether derived from NOEC or EC10 values, are not “certain.” Small deviations between tests using the same organisms and substances in different laboratories are common and only to be expected in view of stochasticity in biological (and environmental fate) processes (e.g., Hamda et al. 2014; Wender et al. 2018). In addition, although an assessment factor of 10 is commonly used for chronic PNEC derivation, this factor could potentially be higher or lower, depending on properties of the test substance in question, specifically on the depth of knowledge of its mode of action and the presence or absence of the respective biological receptors in the different organism groups. Hence, even the figure of <6% at risk (see just above) must be taken with a good measure of scepticism—the fraction at risk could be higher or lower.

Last but not least, the dearth or absence of feasible alternatives to MPM/MPA for immune‐challenged patients must be addressed as well. Only a limited number of tested immunosuppressive APIs is available for solid‐organ transplant patients, or, off‐label, for certain autoimmune diseases. Taylor et al. (2005) and the World Health Organization (2019) list several small‐molecule alternatives to MPA for solid‐organ transplant patients, in addition to the more recent biologics (poly‐ or monoclonal antibodies) that all need to be administered intravenously. However, these small molecules cannot be used or exchanged indiscriminately due to different use, metabolism, and adverse effects profiles (Taylor et al. 2005; Straub 2016). Hence, restricting proven, efficacious APIs is available for these patient groups might have extremely serious consequences. Thus, patient benefit and the dearth of alternative APIs may be in opposition to environmental safety in a number of cases. How can this contradiction be resolved? It may boil down to the question, “What can be done to minimize MPA concentrations in the environment?” We are well aware that the following considerations clearly go beyond pure risk assessment, but we still want to list some ideas. Risk reduction measures aimed at lowering PECs are scarce for human medicines, specifically for APIs like MPM/MPA for which noncompliance is not an issue. In such cases, providing or upgrading to the best available technology in STPs will certainly play a highly important role (e.g., Abegglen and Siegrist, 2012; Björlenius and Breitholz 2016), in particular with a substance that basically is biodegradable like MPA. The situation may be somewhat different in cases in which alternative APIs with the same therapeutic function exist, but even in such cases substitution should be applied with caution (Straub 2016). In addition, in view of the limited oral bioavailability of MPA itself, the administration of the prodrug MPM could ensure active concentrations of MPA in the patients at lower overall dosages, thanks to the higher bioavailability of MPM; this would consequently decrease the amount of MPA excreted into wastewaters and ultimately into the environment (Straub 2016). Both are potential measures to reduce the worst‐case concentrations of MPA in the environment, without denying a potentially life‐saving medicine to anyone. As maintained by patient organizations, the pharmaceutical industry, and the European Commission (2019), patient access to medicines must not be compromised.

## CONCLUSIONS

Both MPA and its prodrug MPM are excreted as MPA and its glucuronide. In STPs, 12 to 73.1% of total MPA will be biodegraded, whereas adsorption to sludge (with subsequent transfer to soil) is negligible. In the receiving waters, further photo‐ and biodegradation are expected to be important fate processes for MPA. Therefore, the PECs depend mainly on actual use of MPM plus MPA in different countries, on per capita water use, on removal in STPs, on average dilution of the STP effluents in the different states, and on further degradation in the receiving waters. The present study suggests that PEC models of different complexities correspond reasonably well with each other and with the few MECs retrieved. Comparing in particular ePiE PECs with the PNECs results in a risk assessment that is not a trivial “risk” or “no risk” conclusion. For the majority of situations, no significant risk appears, but in a minor fraction there is potential risk with 1 < risk quotient < 10. However, ePiE also identifies river stretches with increased concentrations that are potentially at risk, highlighting point sources (STPs) that might profit from upgrading. Therefore, ePiE emerges as a potent instrument for geographically detailed PEC derivation and environmental risk assessment, and as a guide for risk management, in particular when additional catchments, ideally all through Europe, are integrated. On another level, this differentiated environmental risk assessment of MPA in surface waters calls for a rational, science‐based, societal and political discussion of what kind and what percentage of risk to the environment society is willing or not willing to accept—and conversely, what kind of risk management measures society is willing to impose or install, particularly in the light of unhindered patient access to a life‐saving medicine.

## Supplemental Data

The Supplemental Data are available on the Wiley Online Library at DOI: http://10.1002/etc.4524.

## Conflict of Interest

J.O. Straub, T. Pfister, and A. Häner are full‐time employees of F.Hoffmann‐La Roche in Basle, Switzerland, where they work as an occupational toxicologist (T. Pfister) or environmental risk assessors (J.O. Straub, A. Häner) for Roche. R. Oldenkamp is an independent academic researcher (at Radboud University in the Netherlands and at the University of York in the UK) who has no financial dependency on nor interests in Roche.

## Supporting information

This article includes online‐only Supplemental Data.

Supporting information.

## Data Availability

Final reports for environmental fate and toxicity tests belong to F.Hoffmann‐La Roche (Basle, Switzerland). They may be reviewed in person, on prior request to J.O. Straub (juerg.straub@roche.com) or to A. Häner (andreas.haener.ah1@roche.com), at Roche's site in Basle, without, however, the use of copying, fax, photographic, or mobile phone equipment. The ePiE environmental fate model has been described by Oldenkamp et al. (2018); please contact R. Oldenkamp for access and conditions to use this model (r.oldenkamp@science.ru.nl or r.oldenkamp@gmail.com).

## References

[etc4524-bib-0001] Abegglen C , Siegrist H . 2012 Mikroverunreinigungen aus kommunalem Abwasser. Verfahren zur weitergehenden Elimination auf Kläranlagen. Bundesamt für Umwelt (BAFU), Berne, Switzerland. [cited 2019 June12]. Available from: https://www.bafu.admin.ch/bafu/de/home/themen/wasser/publikationen-studien/publikationen-wasser/mikroverunreinigungen-aus-kommunalem-abwasser.html—English abstract Micropollutants in municipal wastewater (Summary). [cited 2019 June 12]. Available from: https://www.bafu.admin.ch/bafu/en/home/topics/water/water--publications/publications-water/micropollutants-municipal-wastewater-summary.html

[etc4524-bib-0002] American Chemical Society . 2018 SciFinder™ subscription database. Columbus OH, USA. [cited 2018 July 9]. Available from: https://www.cas.org/products/scifinder

[etc4524-bib-0003] Anderson HA , Bracewell JM , Fraser AR , Jones D , Robertson GW , Russell JD . 1988 5‐Hydroxymaltol and mycophenolic acid, secondary metabolites from *Penicillium echinulatum* . Trans Br Mycol Soc 91:649–651.

[etc4524-bib-0004] Aubakirova B , Beisenova R , Boxall ABA . 2017 Prioritisation of pharmaceuticals based on risks to aquatic environments in Kazakhstan. Integr Environ Assess Manag 13:832–839. 28120523 10.1002/ieam.1895

[etc4524-bib-0005] Bengtsson‐Palme J , Larsson DGJ . 2016 Concentrations of antibiotics predicted to select for resistant bacteria: Proposed limits for environmental regulation. Environ Int 86:140–149. 26590482 10.1016/j.envint.2015.10.015

[etc4524-bib-0006] Björlenius B , Breitholz M . 2016 Evaluate removal of high risk APIs through wastewater treatment. In Ruden C, ed, *Identification and Reduction of Environmental Risks Caused by Human Pharmaceuticals* MistraPharma Research 2008–2015, Final Report. MistraPharma Research Program, Stockholm, Sweden, pp 26–37. [cited 2019 June 12]. Available from: https://www.mistrapharma.se/Homepage/Download-File/f/829793/h/cbcc9a293b8c034d94535e16efc9f1f6/MistraPharma+final+report+2008%2B2015_v2

[etc4524-bib-0007] Briggs GG . 1973 A simple relationship between soil adsorption of organic chemicals and their octanol/water partition coefficients. *Proceedings*, 7th British Insecticide and Fungicide Conference of the British Crop Protection Council, November 19–22, 1973, Brighton, UK, pp 83–86.

[etc4524-bib-0008] Caldwell DJ , D'Aco V , Davidson T , Kappler K , Murray‐Smith RJ , Owen SF , Robinson PE , Simon‐Hettich B , Straub JO , Tell J . 2018 Environmental risk assessment of metformin and its transformation product guanylurea: II. Occurrence in surface waters of Europe and the United States and derivation of predicted no‐effect concentrations. Chemosphere 216:855–865. 30385066 10.1016/j.chemosphere.2018.10.038

[etc4524-bib-0009] Campbell PJ , Arnold DJS , Brock TCM , Grandy NJ , Heger W , Heimbach F , Maund SJ , Streloke M , eds, 1999 *Guidance Document on Higher‐tier Aquatic Risk Assessment for Pesticides (HARAP)*. SETAC Europe, Brussels, Belgium.

[etc4524-bib-0010] Center for Drug Evaluation and Research . 1998 Guidance for industry. Environmental assessment of human drug and biologics applications. CMC 6, Revision 1. US Food and Drug Administration, Rockville, MD. [cited 2018 July 4]. Available from: http://www.fda.gov/downloads/Drugs/GuidanceComplianceRegulatoryInformation/Guidances/ucm070561.pdf

[etc4524-bib-0011] Committee for Medicinal Products for Human Use . 2015 Guideline for the environmental risk assessment of medicinal products for human use. EMEA/CHMP/SWP/4447/00 corr 2. European Medicines Agency, London, UK. [cited 2018 July 4]. Available from: http://www.ema.europa.eu/docs/en_GB/Fdocument_library/Scientific_guideline/2009/10/WC500003978.pdf

[etc4524-bib-0012] Cline JC , Nelson JD , Gerzon K , Williams RH , Delong DC . 1969 In vitro antiviral activity of mycophenolic acid and its reversal by guanine‐type compounds. Appl Microbiol 18:14–20. 4308535 10.1128/am.18.1.14-20.1969 PMC377874

[etc4524-bib-0013] Darbra RM , Eljarrat E , Barceló D . 2008 How to measure uncertainties in environmental risk assessment. Trends Anal Chem 27:377–385.

[etc4524-bib-0014] Daouk S , Chèvre N , Vernaz N , Bonnabry P , Dayer P , Daali Y , Fleury‐Souverain S . 2015 Prioritization methodology for the monitoring of active pharmaceutical ingredients in hospital effluents. J Environ Manag 160:324–332. 10.1016/j.jenvman.2015.06.037 26144564

[etc4524-bib-0015] Digits JA , Hedstrom L . 1999 Species‐specific inhibition of inosine 5′‐monophosphate dehydrogenase by mycophenolic acid. Biochemistry 38:15388–15397. 10563825 10.1021/bi991558q

[etc4524-bib-0016] Ďuračková Z , Betina V , Horníková B , Nemec P . 1977 Toxicity of mycotoxins and other fungal metabolites to *Artemia salina* larvae. Zbl Bakt Abt II 132:294–299. 10.1016/s0044-4057(77)80017-8 910570

[etc4524-bib-0017] Eckardt K , Stahlmann R . 2010 Use of two validated in vitro tests to assess the embryotoxic potential of mycophenolic acid. Arch Toxicol 84:37–43. 19856175 10.1007/s00204-009-0476-1

[etc4524-bib-0018] European Chemicals Agency . 2018 Registration, Evaluation, Authorisation and Restriction of Chemicals database. Registration dossier for MPA. Helsinki, Finland. [cited 2018 July 13]. Available from: https://www.echa.europa.eu/web/guest/registration-dossier/-/registered-dossier/15977/6/2/6

[etc4524-bib-0019] European Commission . 2011 Common implementation strategy for the Water Framework Directive. 2000/60/EC. Guidance document 27: Technical guidance for deriving environmental quality standards. EC Technical Report 2011–055. Brussels, Belgium. [cited 2018 July 6]. Available from: https://circabc.europa.eu/sd/a/0cc3581b-5f65-4b6f-91c6-433a1e947838/TGD-EQS%20CIS-WFD%2027%20EC%202011.pdf

[etc4524-bib-0020] European Commission . 2018 Eurostat. Population on 1 January, Table. European Statistics Office, Brussels, Belgium. [cited 2018 July 6]. Available from: http://ec.europa.eu/eurostat/tgm/table.do?tab=table&init=1&language=en&pcode=tps00001&plugin=1

[etc4524-bib-0021] European Commission . 2019 European strategic approach to pharmaceuticals in the environment. Communication from the Commission to the European Parliament, the Council and the European Economic and Social Committee. COM(2019) 128 final; Brussels, Belgium, 11.3.2019. [cited 2019 March 12]. Available from: http://ec.europa.eu/environment/water/water-dangersub/pdf/strategic_approach_pharmaceuticals_env.PDF

[etc4524-bib-0022] European Medicines Agency . 2018 Guideline on the environmental risk assessment of medicinal products for human use. Draft. EMEA/CHMP/SWP/4447/00 Rev. 1, 15 November 2018. Safety Working Party, London, UK. [cited 2018 December 10]. Available from: https://www.ema.europa.eu/documents/scientific-guideline/draft-guideline-environmental-risk-assessment-medicinal-products-human-use-revision-1_en.pdf

[etc4524-bib-0023] FASS . 2018 Swedish medicines database. Läkemedelsindustriföreningen, Stockholm, Sweden. [cited 2018 July 13]. Available from: http://www.fass.se/LIF/product?userType=0&nplId=20040514000022

[etc4524-bib-0024] Florey HW , Gilliver K , Jennings MA , Sanders AG . 1946 Mycophenolic acid, an antibiotic from *Penicillium brevicompactum* Dierckx. Lancet Jan 12, 1946:46–49. 10.1016/s0140-6736(46)90242-5 21010114

[etc4524-bib-0025] Franquet‐Griell H , Gómez‐Canela C , Ventura F , Lacorte S . 2015 Predicting concentrations of cytostatic drugs in sewage effluents and surface waters of Catalonia (NE Spain). Environ Res 138:161–172. 25721243 10.1016/j.envres.2015.02.015

[etc4524-bib-0026] Franquet‐Griell H , Ventura F , Boleda MR , Lacorte S . 2016 Do cytostatic drugs reach drinking water? The case of mycophenolic acid. Environ Pollut 208:532–536. 26552545 10.1016/j.envpol.2015.10.026

[etc4524-bib-0027] Franquet‐Griell H , Medina A , Sans C , Lacorte S . 2017a Biological and photochemical degradation of cytostatic drugs under laboratory conditions. J Hazard Mater 323:319–328. 27421981 10.1016/j.jhazmat.2016.06.057

[etc4524-bib-0028] Franquet‐Griell H , Pueyo V , Silva J , Orera VM , Lacorte S . 2017b Development of a macroporous ceramic passive sampler for the monitoring of cytostatic drugs in water. Chemosphere 182:681–690. 28528314 10.1016/j.chemosphere.2017.05.051

[etc4524-bib-0029] Franquet‐Griell H , Cornadó D , Caixach J , Ventura F , Lacorte S . 2017c Determination of cytostatic drugs in Besòs River (NE Spain) and comparison with predicted environmental concentrations. Environ Sci Pollut Res 24:6492–6503. 10.1007/s11356-016-8337-y 28074365

[etc4524-bib-0030] Fulton B , Markham A . 1996 Mycophenolate mofetil. A review of its pharmacodynamic and pharmacokinetic properties and clinical efficacy in renal transplantation. Drugs 51:278–298. 8808168 10.2165/00003495-199651020-00007

[etc4524-bib-0031] Gao X‐P , Feng F , Zhang X‐Q , Liu X‐X , Wang Y‐B , She J‐X , He Z‐H , He M‐F . 2014 Toxicity assessment of 7 anticancer compounds in zebrafish. Int J Toxicol 33:98–105. 24563414 10.1177/1091581814523142

[etc4524-bib-0032] Giebułtowicz J , Nałęcz‐Jawecki G . 2016 Occurrence of immunosuppressive drugs and their metabolites in the sewage‐impacted Vistula and Utrata Rivers and in tap water from the Warsaw region (Poland). Chemosphere 148:137–147. 26803579 10.1016/j.chemosphere.2015.12.135

[etc4524-bib-0033] Gunnarsson L , Jauhiainen A , Kristiansson E , Nerman O , Larsson DGJ . 2008 Evolutionary conservation of human drug targets in organisms used for environmental risk assessments. Environ Sci Technol 42:5807–5813. 18754513 10.1021/es8005173

[etc4524-bib-0034] Guo J , Sinclair CJ , Selby K , Boxall ABA . 2015 Toxicological and ecotoxicological risk‐based prioritization of pharmaceuticals in the natural environment. Environ Toxicol Chem 35:1550–1559. 10.1002/etc.3319 26799673

[etc4524-bib-0035] Hamda NT , Forbes VE , Stark JD , Laskowski R . 2014 Stochastic density‐dependent matrix model for extrapolating individual‐level effects of chemicals to the population: Case study on effects of Cd on *Folsomia candida* . Ecol Model 280:53–64.

[etc4524-bib-0036] Hart A , ed. 2001 *Probabilistic Risk Assessment for Pesticides in Europe: Implementation & Research Needs*. Central Science, Laboratory, York, UK.

[etc4524-bib-0037] IQVIA . 2018. IQVIA MIDAS Quantum, Q1 2018 subscription database. Durham, NC, USA.

[etc4524-bib-0038] International Union for Conservation of Nature . 2019 Distribution map of the European otter. Gland, Switzerland. [cited 2019 January 1]. Available from http://maps.iucnredlist.org/map.html?id=12419

[etc4524-bib-0092] iPiE . 2019 Intelligence‐led assessment of pharmaceuticals in the environment. [cited 2019 January 4]. Accessed from: http://i-pie.org/

[etc4524-bib-0039] Japanese Ministry of Health, Labor, and Welfare . 2016, Draft guidance on environmental impact assessment of new drugs. PSEHB/ELD Notification No. 0330‐1, dated March 30, 2016. The Director of Evaluation and Licensing Division, Pharmaceutical Safety and Environmental Health Bureau, Ministry of Health, Labour and Welfare, Tokyo, Japan.

[etc4524-bib-0040] Keller VDJ , Williams RJ , Lofthouse C , Johnson AC . 2014 Worldwide estimation of river concentrations of any chemical originating from sewage‐treatment plants using dilution factors. Environ Toxicol Chem 33:447–452. 24375744 10.1002/etc.2441 PMC4253128

[etc4524-bib-0041] Kooijman SALM . 1981 Parametric analyses of mortality rates in bioassays. Water Res 15:107–119.

[etc4524-bib-0042] Kümmerer K . 2016 Presence, fate and risks of pharmaceuticals in the environment In Summerton L, Sneddon HF, Jones LC, Clark JH, eds, Green and Sustainable Medicinal Chemistry: Methods, Tools and Strategies for the 21st Century Pharmaceutical Industry. Royal Society of Chemistry, London, UK *Green Chem* 46:63–72.

[etc4524-bib-0043] Kümmerer K , Henninger A . 2003 Promoting resistance by the emission of antibiotics from hospitals and households into effluent. Clin Microbiol Infect Dis 9:1203–1214. 10.1111/j.1469-0691.2003.00739.x 14686985

[etc4524-bib-0044] Lee WA , Gu L , Miksztal AR , Chu N , Leung K , Nelson PH . 1990 Bioavailability improvement of mycophenolic acid through amino ester derivatization. Pharm Res 7:161–166. 2308896 10.1023/a:1015828802490

[etc4524-bib-0045] Link M , von der Ohe PC , Voss K , Schafer RB . 2017 Comparison of dilution factors for German wastewater treatment plant effluents in receiving streams to the fixed dilution factor from chemical risk assessment. Sci Total Environ 598:805–813. 28458197 10.1016/j.scitotenv.2017.04.180

[etc4524-bib-0046] Marín‐García M . 2015 Study of photodegradation processes of environmental organic pollutants by UV spectrophotometry, liquid chromatography with UV and MS detection and chemometric methods. Treball Final de Grau (Master's thesis), University of Barcelona, Barcelona, Spain. [cited 2018 July 10]. Available from: https://www.researchgate.net/profile/Marc_Marin-Garcia/publication/313553072_Study_of_photodegradation_processes_of_environmental_organic_pollutants_by_UV_spectrophotometry_liquid_chromatography_with_UV_and_MS_detection_and_chemometric_methods/links/589de012a6fdccf5e96a537d/Study-ofphotodegradation-processes-of-environmental-organicpollutants-by-UV-spectrophotometry-liquidchromatography-with-UV-and-MS-detection-andchemometric-methods.pdf

[etc4524-bib-0047] McCall PJ , Swann RL , Laskowski DA , Unger SM , Vrona SA , Dishburger HJ . 1980 Estimation of chemical mobility in soil from liquid chromatographic retention times. Bull Environ Contam Toxicol 24:190–195. 7362897 10.1007/BF01608096

[etc4524-bib-0048] Möhle E , Metzger JW . 2001 Drugs in municipal sewage effluents: Screening and biodegradation studies. In Daughton CG, Jones‐Lepp TL, eds, *Pharmaceuticals and Personal Care Products in the Environment; Scientific and Regulatory Issues*. ACS Symposium Series 791. American Chemical Society, Washington DC, USA, pp 192–205.

[etc4524-bib-0049] Murray‐Smith RJ , Coombe VT , Haag Grönlund M , Waern F , Baird JA . 2012 Managing emissions of active pharmaceutical ingredients from manufacturing facilities: An environmental quality standard approach. Integr Environ Assess Manag 8:320–330. 22057894 10.1002/ieam.1268

[etc4524-bib-0050] Noto T , Sawada M , Ando K , Koyama K . 1969 Some biological properties of mycophenolic acid. J Antibiot 12:165–169. 10.7164/antibiotics.22.165 5800425

[etc4524-bib-0051] Organisation for Economic Co‐operation and Development . 1984 Test No. 202: *Daphnia* sp. acute immobilisation test and reproduction test. *OECD Guidelines for the Testing of Chemicals* Paris, France. [cited 2018 July 15]. Available from: https://www.oecd-ilibrary.org/environment/oecd-guidelines-for-the-testing-of-chemicals-section-2-effects-on-biotic-systems_20745761

[etc4524-bib-0052] Organisation for Economic Co‐operation and Development . 1992 Test No. 301F: Biodegradation test—O2 consumption. *OECD Guidelines for the Testing of Chemicals*. Paris, France.

[etc4524-bib-0053] Organisation for Economic Co‐operation and Development . 1993 Test No. 203: Fish, acute toxicity test. *OECD Guidelines for the Testing of Chemicals* Paris, France. [cited 2018 July 15]. Available from: https://www.oecd-ilibrary.org/environment/oecd-guidelines-for-thetesting-of-chemicals-section-2-effects-on-biotic-systems_20745761

[etc4524-bib-0054] Organisation for Economic Co‐operation and Development . 2000 Test No. 106: Adsorption–desorption using a batch equilibrium method. *OECD Guidelines for the Testing of Chemicals* Paris, France. [cited 2018 July 15]. Available from: https://www.oecd-ilibrary.org/environment/oecd-guidelines-for-the-testing-of-chemicals-section-1-physical-chemical-properties_20745753

[etc4524-bib-0055] Organisation for Economic Co‐operation and Development . 2003 Draft guidance document for the statistical analysis of ecotoxicity data. OECD Environmental Health and Safety Publications, Series on Testing and Assessment. Paris, France. [cited 2018 December 10]. Available from: http://www.oecd.org/chemicalsafety/testing/2956192.pdf

[etc4524-bib-0056] Organisation for Economic Co‐operation and Development . 2008 Test No. 314B: Biodegradation in activated sludge. *OECD Guidelines for the Testing of Chemicals* Paris, France. [cited 2018 July 15]. Available from: https://www.oecd-ilibrary.org/environment/oecd-guidelines-for-the-testing-of-chemicals-section-3-degradation-and-accumulation_2074577x

[etc4524-bib-0057] Organisation for Economic Co‐operation and Development . 2010 Test No. 209: Activated sludge, respiration inhibition test. *OECD Guidelines for the Testing of Chemicals* Paris, France. [cited 2018 July 15]. Available from: https://www.oecd-ilibrary.org/environment/oecd-guidelines-for-the-testing-of-chemicals-section-2-effects-on-biotic-systems_20745761

[etc4524-bib-0058] Organisation for Economic Co‐operation and Development . 2011 Test No. 201: Freshwater alga and cyanobacteria, growth inhibition test. *OECD Guidelines for the Testing of Chemicals* Paris, France. [cited 2018 July 15]. Available from: https://www.oecd-ilibrary.org/environment/oecd-guidelines-for-the-testing-of-chemicals-section-2-effects-on-biotic-systems_20745761

[etc4524-bib-0059] Organisation for Economic Co‐operation and Development . 2012a Test No. 211: *Daphnia magna* reproduction test. *OECD Guidelines for the Testing of Chemicals* Paris, France. [cited 2018 July 15]. Available from: https://www.oecd-ilibrary.org/environment/oecd-guidelines-for-the-testing-of-chemicals-section-2-effects-on-biotic-systems_20745761

[etc4524-bib-0060] Organisation for Economic Co‐operation and Development . 2012b Test No. 229: Fish short term reproduction assay. *OECD Guidelines for the Testing of Chemicals* Paris, France. [cited 2018 July 15]. Available from: https://www.oecd-ilibrary.org/environment/oecd-guidelines-for-the-testing-of-chemicals-section-2-effects-on-biotic-systems_20745761

[etc4524-bib-0061] Organisation for Economic Co‐operation and Development . 2013 Test No. 210: Fish, early‐life stage toxicity test. *OECD Guidelines for the Testing of Chemicals* Paris, France. [cited 2018 July 15]. Available from: https://www.oecd-ilibrary.org/environment/oecd-guidelines-for-the-testing-of-chemicals-section-2-effects-on-biotic-systems_20745761

[etc4524-bib-0062] Organisation for Economic Co‐operation and Development . 2018 *OECD Guidelines for the Testing of Chemicals* Paris, France. [cited 2018 July 4]. Available from: https://www.oecd-ilibrary.org/environment/oecd-guidelines-for-the-testing-of-chemicals-section-1-physical-chemical-properties_20745753

[etc4524-bib-0063] Oldenkamp R , Hoeks S , Čengić M , Barbarossa V , Burns EE , Boxall ABA , Ragas AMJ . 2018 A high‐resolution spatial model to predict exposure to pharmaceuticals in European surface waters: ePiE. Environ Sci Technol 52:12494–12503. 30303372 10.1021/acs.est.8b03862 PMC6328286

[etc4524-bib-0064] Roche . 2017a Safety data sheet for mycophenolic acid. F.Hoffmann‐La Roche, Basle, Switzerland. [cited 2018 April 1]. Available from: https://www.roche.com/sustainability/environment/safety_data_sheets-row.htm

[etc4524-bib-0065] Roche . 2017b Safety data sheet for mycophenolate mofetil. F.Hoffmann‐La Roche, Basle, Switzerland. [cited 2018 April 1]. Available from: https://www.roche.com/sustainability/environment/safety_data_sheets-row.htm

[etc4524-bib-0066] Roos V , Gunnarsson L , Fick J , Larsson DG , Rudén C . 2012 Prioritising pharmaceuticals for environmental risk assessment: Towards adequate and feasible first‐tier selection. Sci Total Environ 421–422:102–110. 10.1016/j.scitotenv.2012.01.039 22361586

[etc4524-bib-0067] Rossi L , Cheseaux L . 2013 Sources diffuses de micropolluants dans le Léman: Etude de bassins versants spécifiques et définition d'outils d’extrapolation. Rapport d’étude de l’EPFL, laboratoire de technologie écologique (ECOL), sur mandat de l’Office fédéral de l’environnement (OFEV). EPF Lausanne, Switzerland, 101 p + Annexes. [cited 2018 July 23]. Available from: https://infoscience.epfl.ch/record/186688

[etc4524-bib-0068] Royal Pharmaceutical Society . 2018 MedicinesComplete subscription database. Clarke's analysis of drugs and poisons, mycophenolate mofetil. London, UK. [cited 2018 July 4]. Available from: https://www.medicinescomplete.com/#/content/clarke/CLK1128#d1e492587

[etc4524-bib-0069] Santos MSF , Franquet‐Griell H , Lacorte S , Madeira LM , Alves A . 2017a Anticancer drugs in Portuguese surface waters—Estimation of concentrations and identification of potential priority drugs. Chemosphere 184:1250–1260. 28672724 10.1016/j.chemosphere.2017.06.102

[etc4524-bib-0070] Santos R , Ursu O , Gaulton A , Patrícia Bento A , Donadi RS , Bologa CG , Karlsson A , Al‐Lazikani B , Hersey A , Oprea TI , Overington JP . 2017b A comprehensive map of molecular drug targets. Nature Rev Drug Disc 16:19–34. 10.1038/nrd.2016.230 PMC6314433 27910877

[etc4524-bib-0071] Shipkova M , Armstrong VW , Oellerich M , Wieland E . 2005 Mycophenolate mofetil in organ transplantation: Focus on metabolism, safety and tolerability. Expert Opin Drug Metab Toxicol 1:505–526. 16863458 10.1517/17425255.1.3.505

[etc4524-bib-0072] Silverman Kitchin JE , Keltz Pomeranz M , Pak G , Washenik K , Shupack JL . 1997 Rediscovering mycophenolic acid: A review of its mechanism, side effects, and potential uses. J Am Acad Dermatol 37:445–449. 9308561 10.1016/s0190-9622(97)70147-6

[etc4524-bib-0073] Singer HP , Wössner AE , McArdell CS , Fenner K . 2016 Rapid screening for exposure to “non‐target” pharmaceuticals from wastewater effluents by combining HRMS‐based suspect screening and exposure modeling. Environ Sci Technol 50:6698–6707. 26938046 10.1021/acs.est.5b03332

[etc4524-bib-0074] Straub JO . 2008 Deterministic and probabilistic environmental risk assessment for diazepam In Kümmerer K, ed, Pharmaceuticals in the Environment: Sources, Fate, Effects and Risks, 3rd ed. Springer, Heidelberg, Germany, pp 343–383.

[etc4524-bib-0075] Straub JO . 2016 Reduction in the environmental exposure of pharmaceuticals through diagnostics, Personalised Healthcare and other approaches. A mini review and discussion paper. Sust Chem Pharm 3:1–7.

[etc4524-bib-0076] Straub JO , Hutchinson TH . 2014 Environmental risk assessment for human pharmaceuticals: The current state of international regulations In Brooks BW, Huggett DW, eds, Human Pharmaceuticals in the Environment: Current and Future Perspectives. Emerging Topics in Ecotoxicology 4. Springer, New York, NY, USA, pp 17–47.

[etc4524-bib-0077] Struijs J . 2015 Application of SimpleTreat 4.0 in European substance regulations. UBA Texte 13/2015. Umweltbundesamt, Dessau, Germany. [cited 2018 July 6]. Available from: https://www.umweltbundesamt.de/en/publikationen/application-of-simpletreat-40-in-european-substance. SimpleTreat 4 model: Available from: https://www.rivm.nl/en/Topics/S/Soil_and_water/SimpleTreat/SimpleTreat_4_0_download_form

[etc4524-bib-0078] Suter GW . 1990 Uncertainty in environmental risk assessment. In von Furstenberg GM, ed, *Acting under Uncertainty: Multidisciplinary Conceptions*. Theory and Decision Library, Series A: Philosophy and Methodology of the Social Sciences, Vol 13. Springer, Dordrecht, The Netherlands, pp 203–230.

[etc4524-bib-0079] Taylor AL , Watson CJE , Bradley JA . 2005 Immunosuppressive agents in solid organ transplantation: Mechanisms of action and therapeutic efficacy. Crit Rev Oncol/Hematol 56:23–46. 10.1016/j.critrevonc.2005.03.012 16039869

[etc4524-bib-0080] Uehlinger U , Arndt H , Wantzen KM , Leuven RSEW . 2009 The Rhine River Basin. In: Tockner K, Uehlinger U, Robinson CT, eds, *Rivers of Europe*. Academic, Cambridge, MA, USA, pp 199–245.

[etc4524-bib-0081] US Environmental Protection Agency . 2016 EPISuite: Estimation Programs Interface Suite™ for Microsoft Windows, Ver 4.11. Washington, DC. [cited 2018 July 9]. Available from: http://www.epa.gov/oppt/exposure/pubs/episuite.htm

[etc4524-bib-0082] US Food and Drug Administration . 2006 *Daphnia acute toxicity*. Washington, DC.

[etc4524-bib-0083] van der Hoeven N , Noppert F , Leopold A . 1997 How to measure no effect. Part I: Towards a new measure of chronic toxicity in ecotoxicology. Introduction and workshop results. Environmetrics 8:241–248.

[etc4524-bib-0084] Verbruggen B , Gunnarsson L , Kristiansson E , Österlung T , Owen SF , Snape JR , Tyler CR . 2017 ECOdrug: A database connecting drugs and conservation of their targets across species. Nucl Acids Res. 10.1093/nar/gkx1024. [cited 2018 July 4]. Available from: http://www.ecodrug.org/ PMC5753218 29140522

[etc4524-bib-0085] Veselý D , Veselá D . 1991 Use of chick embryos for prediction of embryotoxic effects of mycotoxins in mammals. Veterinarni Medicina 36:175–181. PMID:1746066. [cited 2018 July 25]. Available from: http://europepmc.org/abstract/MED/1746099 1746099

[etc4524-bib-0086] Wender BA , Prado V , Fantke P , Ravikumar D , Seager TP . 2018 Sensitivity‐based research prioritization through stochastic characterization modeling. Int J Life Cycle Assess 23:324–332.

[etc4524-bib-0087] World Health Organization . 2019 ATC/DDD Index 2019. WHO Collaborating Centre for Drug Statistics Methodology, Norwegian Institute of Public Health, Oslo, Norway. [cited 2019 June 12]. Available from: https://www.whocc.no/atc_ddd_index/?code=L04A

[etc4524-bib-0088] Wilson R , Crouch EAC . 1987 Risk assessment and comparisons: An introduction. Science 236:267–280. 3563505 10.1126/science.3563505

[etc4524-bib-0089] Wishart DS , Feunang YD , Guo AC , Lo EJ , Marcu A , Grant JR , Sajed T , Johnson D , Li C , Sayeeda Z , Assempour N , Iynkkaran I , Liu Y , Maciejewski A , Gale N , Wilson A , Chin L , Cummings R , Le D , Pon A , Knox C , Wilson M . 2018 DrugBank 5.0: A major update to the DrugBank database for 2018. Nucleic Acids Res 46:D1074–D1082. [cited 2018 July 4]. Available from: https://www.drugbank.ca/drugs/DB01024 29126136 10.1093/nar/gkx1037 PMC5753335

[etc4524-bib-0090] Wright JM . 1951 Phytotoxic effects of some antibiotics. Ann Bot 15:493–499.

[etc4524-bib-0091] Wu X , Zhong H , Song J , Damoiseaux R , Yang Z , Lin S . 2006 Mycophenolic acid is a potent inhibitor of angiogenesis. Arterioscler Thromb Vasc Biol 26:2414–2416. 16990565 10.1161/01.ATV.0000238361.07225.fc

