# Supplementary material for: Environmental Risk Assessment for the Active Pharmaceutical Ingredient Mycophenolic Acid in European Surface Waters
Source: Environ Toxicol Chem. 2019 Sep 19;38(10):2259–78. doi: 10.1002/etc.4524 (PMC6856805; doi:10.1002/etc.4524)
Supplement: Supplementary file 1 — Supporting information. [file ETC-38-2259-s001.docx]

**Supplemental Information**

Environmental Risk Assessment for the Active Pharmaceutical Ingredient Mycophenolic Acid in European Surface Waters

Jürg Oliver Straub, Rik Oldenkamp, Thomas Pfister, Andreas Häner

1. **Testing protocols**
   1. The vapour pressure of MPA was estimated by Syntex by comparing losses of MPA under a nitrogen stream with losses in capped vials and comparing the results with the same losses of a reference substance of known vapour pressure (Young & Licato 1994a).
   2. A screening hydolysis test was performed by Syntex with MPA in 0.1 M buffered solutions in triplicate at pH 5, 7 and 9 over 5 days at 50 °C, with HPLC determination of MPA at the start and end of the exposure, 'closely following US Food and Drug Administration 1987 Environmental Assessment Technical Assistance Handbook recommendations' (Young & Licato 1994b).
   3. The photodegradation of MPA in buffered aqueous solutions at pH 5, 7 and 9 under natural sunlight was determined by Syntex, 'closely following the US FDA guideline' (Nicholson 1994)). MPA was used in solutions of 3.12×10^–5^ M (= 10 mg/L); the pH 5 solution was buffered with 0.1 M sodium acetate, the pH 7 solution with 0.1 M monobasic sodium phosphate and the pH 9 solution with 0.1 M sodium borate buffer. As actinometers (reference photodegradants) p-nitroanisole at 1.00×10^–5^ M was used, with 4.96×10^–3^ M pyridine for pH 5, with 6.20×10^–3^ M pyridine for pH 7 and with 9.93×10^–3^ M pyridine for pH 9. In each of the seasonal studies, hermetically closed quartz tubes with either one of the MPA solutions or the corresponding actinometer solutions were exposed to natural sunlight in triplicate. Single control tubes containing either MPA solution or actinometer solution, both at different pHs, were wrapped in aluminium foil and covered in black plastic and exposed in parallel. The tubes were set up in Palo Alto, Ca, USA at 37° 25' N, in parallel on a board painted black, which was exposed on a stand pointing due North at an angle of 30°. Data for the the four seasonal experiments, with date, time of initiation and end, sampling intervals, weather, cloud cover and temperature, are given in the report [4]. At each of several equivalent timepoints, one set of tubes was removed from sunlight, analysed by HPLC and compared with the respective dark control. The substance loss of p-nitroanisole, with known quantum yield, in the control tubes served to calibrate the loss of MPA in the test tubes and determine the photolytic rate constants k_photo_ and half-lives for MPA at different pH levels and insolations over the seasons.
   4. A river sediment/water fate test with two different ^14^C-MPMs (one carboxyl-^14^C-labelled, the other morpholine-^14^C-labelled) was commissioned in 1994 by Syntex according to US FDA Guideline 3.11 in compliance with GLP (Yan 1995). Both carboxyl-^14^C-labelled and morpholine-^14^C-labelled MPM were tested separately for biodegradability in river water and sediment at dosing concentrations of 1 and 5 mg MPM/L, in parallel test set-ups in the dark at 21±1 °C over 64 days. Air was drawn in flow-through systems first through a 2 N KOH vessel to remove natural CO_2_, then through the test vessel and subsequently through two sequential 2 N KOH traps for ^14^CO_2_ capture. Samples from the latter traps were taken for duplicate ^14^CO_2_ analysis by LSC on days 1, 2, 3, 7, 14, 21, 28, 35, 49 and 64. Test medium samples for HPLC analysis were taken on days 0.01 (15 min after dosing), 1, 3, 7, 14, 21, 35, 49 and 64. Sediment samples were taken at the end for combustion and ^14^CO_2_ analysis.
   5. An OECD 106 phase I batch equilibrium adsorption test was performed by Toxi-Coop, Balatonfűred, Hungary (Halász-Laky 2018) with MPA and three soils plus two activated sludges of different characteristics (*p*H_KCl_, organic carbon (OC) content, plus for the soils sand fraction, silt fraction, clay fraction and USDA soil texture).

| Soil/  Substrate | *p*H(KCl) | Organic Carbon, % | Sand, % | Silt, % | Clay, % | Soil texture (USDA classes) |
| --- | --- | --- | --- | --- | --- | --- |
| I 30 02 1 | 6.63 | 2.98 | 2.5 | 61.7 | 35.9 | silty clay loam |
| I 21 02 1 | 4.30 | 0.51 | 59.3 | 25.5 | 15.3 | sandy loam |
| I 27 02 1 | 7.04 | 0.69 | 6.7 | 61.9 | 31.5 | siltyclay loam |
| Sludge 1 | 6.63 | 34 | – | – | – | domestic sludge |
| Sludge 2 | 6.46 | 31 | – | – | – | mixed industrial/ domestic sludge |

Soil identifications are supported by GPS co-ordinates for the sampling points. Sludge 1 was from Balatonfüred Wastewater Treatment Plant, Sludge 2 from Balatonfűzfő Wastewater Treatment Plant. USDA classes = soil classification following United States Department of Agriculture.

In short, the substrates were put in contact with MPA in aqueous 0.01 M CaCl_2_ solutions; the mixture was agitated for an appropriate time to reach equilibrium, in the case of MPA for 10 hours. The soil/sludge suspensions were then separated and the aqueous phase diluted twofold with the mobile HPLC phase, analysed by HPLC (Supplemental Information) and the amount of test item in the water phase calculated. The soil or sludge phases were extracted twice with acetonitrile/water/1 mg/mL NaOH solution (75/75/2, v/v/v); the extracts were diluted twofold with the mobile phase and then analysed by HPLC. Based on the analytics the mass balance could be established. The adsorption constant K_d_ was calculated as MPA concentration adsorbed to substrate divided by MPA concentration in aqueous CaCl_2_ solution; subsequently the K_d_ was normalised to the OC content to result in the K_OC_ for the different substrates (Halász-Laky 2018).

- 1. An OECD 314 B biodegradation simulation test was performed by ECT Oekotoxikologie with carboxyl-^14^C-radiolabelled MPA (radiochemical purity: >98%, specific activity: 47.7 mCi/mmol) over 28 days at 20–25 °C, controlled at ±2 °C (Junker & Herrchen 2017). For the biotic systems, 3 parallel closed 2-L test vessels with a volume of 1518 ml (nominal) activated sludge (AS) from a mainly domestic sewage treatment plant (STP) at 3965 mg dry matter/l starting concentration each were set up. The systems were equipped with a CO_2_-free air flow-through system with KOH and polyethylenglycol traps for inorganic ^14^CO_2_ and organic volatiles, respectively. Two systems were set up with 20 μg ^14^C-MPA/L (corresponding to 6580 dpm/mL) in the actual biotic substance test vessels, the third with solvent control. In addition, a total of 6 abiotic systems (plus one reserve vessel) was set up with 212.6 ml (nominal) activated sludge and 20 μg ^14^C-MPA/L. The systems were sampled and extracted (liquor extract, solids extract, CO_2_ dissolved, CO_2_ evolved (traps)) on a predetermined schedule. The quantification of radioactivity associated with the parent compound was made using thin-layer chromatography (TLC). In addition, the level of parent MPA and potential degradation products was determined in liquor extracts using chromatographic separation and comparison with reference standards where available as well as using radioanalytical detection methods.
  2. An OECD 201 algal growth inhibition test was performed by ECT Oekotoxikologie, Flörsheim, Germany, with the cyanobacterian *Anabaena flos-aquae* with a series of concentrations of MPA in cyanobacteria test medium in different concentrations plus medium-only controls under continuous lighting over 72 h (Gilberg & Chambers 2017). The growth of the algae was measured by calibrated fluorescence intensity of sonicated test solution samples using a fluorometer after 24, 48 and 72 hours. The test concentrations were analysed by HPLC at the start and end of the test. No observed effect concentrations (NOECs and 50% and 10% effective concentrations (EC50, EC10) for yield and growth rate as per the guideline were determined by comparison with the controls and the geometric mean of the measured concentrations (GMMC) by applying statistical tests to the series of raw data (Probit analysis; Shapiro-Wilk's test on normal distribution; Levene's test on variance homogeneity; Williams' multiple sequential t-test). For the algal tests, the NOEC, EC10 and EC50 referring to the growth rate were used as the endpoints.
  3. An OECD 211 chronic reproduction test with the cladoceran *Daphnia magna* was performed by ECT Oekotoxikologie with a series of concentrations of MPA in daphnid test medium plus medium-only controls over 21 days (Egeler & Chambers 2018). The test medium was exchanged every 2–3 days, the daphnids were fed with live algae *(Desmodesmus subspicatus)* throughout the exposure. Fresh and old medium samples were analysed by LC–MS/MS (Supplemental Information) and the time-weighted mean measured concentrations (TWMMC) for the concentrations determined. Statistical data evaluation was performed using Williams's test, Fisher's Exact test, Multiple sequentially rejective U-test after Bonferroni-Holm and Probit analysis. The endpoints in this test were survival and size (length) of the parental daphnids at the end of the test; number of young daphnids released per surviving F0 mother; any malformations or undeveloped eggs; and the intrinsic rate of reproduction (IRR), which integrates both survival and reproduction, as a measure of longer-term stability (IRR ~1), growth (IRR >1) or decline (IRR <1) of a population.
  4. In view of the mammalian mutagenicity and reprotoxicity, a fish partial life-cycle test (PLC; an OECD 229 short-term reproduction test followed by an OECD 210 early-life-stage test) was performed by ECT Oekotoxikologie under GLP with a series of concentrations of MPA in a flow-through setting with the zebrafish *Danio rerio* (Gilberg & Chambers 2018). Briefly, mature fish (F0 generation) in breeding groups of 14 fish were exposed to four concentrations of the test item (0.0005, 0.0029, 0.0171 and 0.100 mg MPA/L), in fish media as well as to controls without the test item, in two replicates per treatment including controls, for 23 days in a 14-hours light to 10-hours dark photoperiod (OECD 229 phase). During this time period, reproduction parameters (survival of parents, fecundity, e.g., number of eggs, fertility and embryo viability/hatchability) of zebrafish exposed to the test item were assessed in comparison with the controls to determine the lowest observed effect concentration (LOEC) and the NOEC. After 23 days, newly fertilised eggs of the F1 generation were collected and exposed to the corresponding series of test item concentrations, 20 eggs in four replicates each per treatment including controls, until 30 days post-hatch (OECD 210 phase). Lethal and sublethal effects of the test item on eggs, larvae and juvenile fish exposed to the test item were assessed in comparison with controls to determine the LOEC and the NOEC. Samples were taken and analysed by LC–MS/MS (Supplemental Information) and the GMMC for the concentrations determined. Statistical check were made using Probit analyses, Weibull analyses, 2- and 3-parameter normal CDF, Shapiro-Wilk’s test on normal distribution, Levene’s test on variance homogeneity, Fisher's Exact Binomial test, Chi² 2×2 Table Test, Welsh t-test, Step-down Cochran-Armitage test Procedure, Williams Multiple Sequential t-test Procedure and Dunnett`s Multiple t-test Procedure.

1. **Test validation**
   1. *Activated sludge degradation test, OECD 314 B*

The OECD 314 B activated sludge degradation test (Junker & Herrchen 2017) fulfilled all quality and validity criteria. Specifically, the radioactivity measured at day 0 in the test vessels was 7412.5 dpm/mL (112.7% of nominal; replicate A) and 7457.0 dpm/mL (113.3% of nominal; replicate B), corresponding to a test item concentration of 22.5 and 22.7 μg/L, respectively. The mean recovery per sampling time point relative to nominally applied radioactivity (aR) was between 104.6% aR (day 28) and 114.5% aR (day 2), while the overall mean recovery for all sampling time points was 108.7±4.0% aR. The mean recovery per sampling time point relative to initially recovered radioactivity (iRR) was between 92.6% iRR (day 28) and 101.3% iRR (day 2), while the overall mean recovery for all sampling time points was 96.2±3.5% iRR.

- 1. *Algal growth inhibition test, OECD 201, with cyanobacteria*

The new OECD 201 algal growth inhibition test with the cyanobacterian *A. flos-aquae* (Gilberg & Chambers 2017) fulfilled all biological validity criteria regarding a minimum mean biomass increase in the controls over 72 hours of at least a factor of 16 (found: 196); mean coefficient of variation for section-by-section specific growth rates in the controls of less than 35% (found: 31.7%); and coefficient of variation of average specific growth rates during the test period in replicate control cultures of less than 10% (found: 2.7%).

- 1. *Daphnid reproduction test, OECD 211*

The new OECD 211 reproduction test with *Daphnia magna* (Gilberg & Chambers 2018) fulfilled all biological validity criteria, viz. mortality of the parent (female) animals in the controls of less than 20% (found: 0%) and mean number of live offspring produced per parent animal in the controls surviving at the end of the test of a minimum of 60 (found: 78.7).

*2.3* *Fish partial life-cycle test, OECD 229 followed by OECD 210*

In the fish PLC partial life-cycle test test with *Danio rerio* (Gilberg & Chambers 2018), all biological validation parameters and criteria were met for both phases. In the OECD 229 phase, ≥90% of control fish must survive (found: 96.4%). In the OECD 210 phase, ≥70% of eggs must hatch in the controls (found: 92.5%) and ≥75% of hatched controls must survive untile the end of the test phase (found: 86.5%).

1. **HPLC method**.

*3.1 Apparatus*

*3.1.1 HPLC system:* Shimadzu LC-20 Prominence

CBM-20A, System Controller, No.: L20235277165

LC-20 AD, Solvent Delivery Unit, No.: L20105278264

SIL-20A HT, Autosampler, No.: L20345270614

SPD-20A, UV-VIS Detector, No.: L20135274968

CTO-20A, Column Oven, No.: L20205274314

OGU-20A5R, Degasser, No.: L20705263744

*3.1.2 HPLC Conditions*

Columns: Luna C18(2); 150 × 4.6 mm, 3 μm

Mobile Phase: Acetonitrile/Water/Formic acid

(500/500/1, v/v/v)

Flow: 1.0 ml/min

Injection volume: 20 μl

Detector: 304 nm

Retention time: 4.5±0.5 min

*3.2 Selectivity*

The following control samples were prepared and analysed to determine the specificity of the assay:

− 0.01 M CaCl2

− blank soil and blank sludge

− extracting agent

− extracting agent with the test item

The specificity of the method was examined by analysis of blank formulations.

No interfering components were detected in the control samples.

*2.3 Repeatability*

The repeatability of the HPLC method was verified with two different concentrations (0.1 and 20 μg/ml) of the calibration samples. A series of seven injections was carried out from the same solution of the above samples at two different concentration levels. The CV (coefficient of variation) of peak areas and retention times of the repeated injections was determined. The repeatability of the peak areas was found to be 1.4% for the 0.1 μg/ml solution and 0.04% for the 20 μg/ml solution, demonstrating acceptable instrument precision.

*3.4 Linearity*

The linearity of the analytical method was evaluated in the range of 0.1–20 μg/ml concentrations of MPA. Eight concentration levels were used: 0.1, 0.2, 0.5, 1, 2, 5, 10 and 20 μg/ml. Eight such series were prepared and analysed on eight separate days with three replicate injections from each solution.

The mean of the peak areas versus the concentration showed good linearity in the range of 0.1–20 μg/ml. The calibration graph was calculated applying weighted linear regression. The weighting factor was 1/concentration. The deviation of the recalculated value from the nominal concentration was ≤8.1%, at the LOQ it was ≤12.4%. The coefficients of the determination (r^2^) were 1.000, thus the calibration model fulfils the linearity requirements.

*3.5 Limit of Quantification*

The lowest concentration of the calibration curve, 0.1 μg/ml, is considered to be the quantification limit (LOQ) of the HPLC method.

*3.6 Results of the Method Validation*

Selectivity No interfering component was detected in the blank samples

Repeatability (7 replicates) CV(%): ≤1.4%

Linear range 0.1–20 μg/ml

Limit of Quantification 0.1 μg/ml

Recovery from 0.01 M CaCl2 104% at 0.2 mg/l concentration level

97% at 20 mg/l concentration level

Stability in 0.01 M CaCl2 at least 1 day at room temperature

Recovery from 3 different types of soil 96–107% at 1 mg/g concentration level

94–100% at 200 mg/g concentration level

Recovery from 2 different types of sludge 99–102% at 1 mg/g concentration level

95–96% at 200 mg/g concentration level

Stock solution stability at least 3 days at 5±3 °C

Stability in the autosampler at least 75 hours

1. LC–MS/MS Method
   1. *Apparatus*

*4.1.1 Instruments:*

Pump Agilent 1100 LC Binary Pump &

1290 Binary Pump

Column Oven Agilent 1100 Series & 1290 Series

Autosampler CTC PAL HTS Autosampler

Detector API 5000 & 5500 LC–MS/MS Systems

*4.1.2 HPLC Conditions*

Columns: Phenomenex Gemini 5 µm 110 Å C18

5.0 × 2.0 mm

Column Temperature 55 ºC

Mobile Phase A 10 mM Ammonium Formate (aq)

Mobile Phase B Acetonitrile

Gradient Time [min] %A %B

0 90 10

5 0 100

5.1 90 10

7 90 10

Flow Divert API 5000 API 5500

0🡪1 min: flow to waste 0🡪7 min: flow to MS

1🡪2 min: flow to MS

2🡪7 min, end of run: flow to waste

Wash solvent 1: MeOH

Wash solvent 2: MeOH/H2O (10/90, v/v)

Flow rate: 1 ml/min

Injection volume: 40 µL

Approximate RT API 5000: 1.9 min, API 5500: 1.5 min

*4.1.3 MS–MS Conditions*

Ion Source Positive Ion Turbo Spray Ionisation

Curtain Gas 20

Temperature 450 °C

Ion Transfer Voltage 5500 V

Collision Gas Cell 5.0

GS1 Nebuliser Gas API 5000: 40, API 5500: 50 (arbitrary units)

GS2 Turbo Gas 60 (arbitrary units)

Scan Type MRM

Ion Mass Transitions 338🡪207 (m/z) 338🡪303 (m/z)

Dwell Time 150 ms 150 ms

Declustering Potential 70 V 70 V

Entrance Potential 10 V 10 V

Collision Energy 30 V 30 V

Collision Cell Exit Potential 15 V 15 V

*4.2 Calibration and Calculation*

Multi-point calibration curves of the reference item were obtained for Mycophenolic Acid by plotting the concentration in ng/ml versus the peak area. The curve was calculated by the method of least squares linear regression. The detector response was linear for matrix-matched calibration solutions of Mycophenolic Acid over the tested concentration range: 0.1 to 60 ng/ml.

The chromatography data system quantified the concentrations of Mycophenolic Acid in samples by comparison to the calibration curve. The amount of Mycophenolic Acid in a given sample was calculated as follows:

| Amount found [mg/l] = | C × D × Vf |
| --- | --- |
|  | 1000 × Vi |

Where:

C = analyte concentration calculated by chromatography data system

D = dilution factor

Vf = final volume of the sample in ml

Vi = initial volume of the sample in ml.

*4.3. Recovery Efficicancy, Limit of Detection and Limit of Quantitation*

The mean recovery efficiency obtained for the untreated water samples spiked with PA was 99% and was within the required range of 70–110%.

The limit of detection (LOD), based on 3 × noise, is 0.000008 mg test item/L (based on unrounded data).

The limit of quantification (LOQ), based on 10 × noise, is 0.00003 mg test item/L (based on unrounded data).

**References**

Egeler P, Chambers J (2018): Mycophenolic acid: A Study on the Chronic Toxicity to *Daphnia magna* according to the OECD Guideline No. 211, *Daphnia magna* Reproduction Test, adopted 2nd October, 2012. ECT Oekotoxikologie, Flörsheim, Germany, on behalf of F.Hoffmann-La Roche Ltd, Basle, Switzerland; unpublished.

Gilberg D, Chambers G (2017): Mycophenolic Acid: A Study on the Toxicity to Blue-green Algae *(Anabaena flos-aquae)* according to OECD Guideline No. 201, Freshwater Alga and Cyanobacteria, Growth Inhibition Test. ECT Oekotoxikologie, Flörsheim, Germany, on behalf of F.Hoffmann-La Roche Ltd, Basle, Switzerland; unpublished.

Gilberg D, Chambers G (2018): Mycophenolic acid: A Shortened Life-cycle test with Zebrafish – Investigation of Effects on F0-Reproduction, F1-Early-Life Stages and F1-Survival, based on OECD (2008): Detailed Review Paper (DRP) on Fish Life-Cycle Tests. OECD Environment, Health and Safety Publications. Series on Testing and Assessment No. 95, ENV/JM/MONO(2008)22. ECT Oekotoxikologie, Flörsheim, Germany, on behalf of F.Hoffmann-La Roche Ltd, Basle, Switzerland; unpublished

Halász-Laky V (2018): Adsorption Study with Mycophenolic Acid Using the Batch Equilibrium Method. Toxi-Coop ZRT, Balatonfüred, Hungary, on behalf of F.Hoffmann-La Roche Ltd, Basle, Switzerland; unpublished.

Junker T, Herrchen M (2017): Mycophenolic acid, [carboxyl-14C]: A Study on the Biodegradation in Activated Sludge according to OECD Guideline No. 314B, Simulation tests to assess the biodegradability of chemicals discharged in wastewater – Biodegradation in Activated Sludge. ECT Oekotoxikologie, Flörsheim, Germany, on behalf of F.Hoffmann-La Roche Ltd, Basle, Switzerland; unpublished.

Nicholson V (1994): Photolysis of mycophenolic acid in aqueous buffers. Syntex Inc., Palo Alto, USA/F.Hoffmann-La Roche, unpublished.

Yan Z (1995): Aerobic biodegradation of 14C-Mycophenolate mofetil in river water and sediment (metabolism test). US FDA Technical Assistance Document 3.11, Aerobic biodegradation in water (with modifications). ABC Laboratories, Columbia MO, USA, on behalf of Syntex Inc., Palo Alto, USA/F.Hoffmann-La Roche, unpublished.

Young A, Licato N (1994a): Preliminary report. Estimated vapor pressure of mycophenolic acid. Syntex Inc., Palo Alto, USA/F.Hoffmann-La Roche, unpublished.

Young A, Licato N (1994b): Hydrolytic stability of mycophenolic acid in aqueous buffers. Screening test: estimation of hydrolytic potential. Syntex Inc., Palo Alto, USA/F.Hoffmann-La Roche, unpublished.
